# Conformational Control of Donor–Acceptor Molecules Using Non-covalent Interactions

**DOI:** 10.1021/acs.jpca.4c03711

**Published:** 2024-09-17

**Authors:** Shawana Ahmad, Julien Eng, Thomas J. Penfold

**Affiliations:** Chemistry—School of Natural and Environmental Sciences, Newcastle University, Newcastle Upon-Tyne NE1 7RU, U.K.

## Abstract

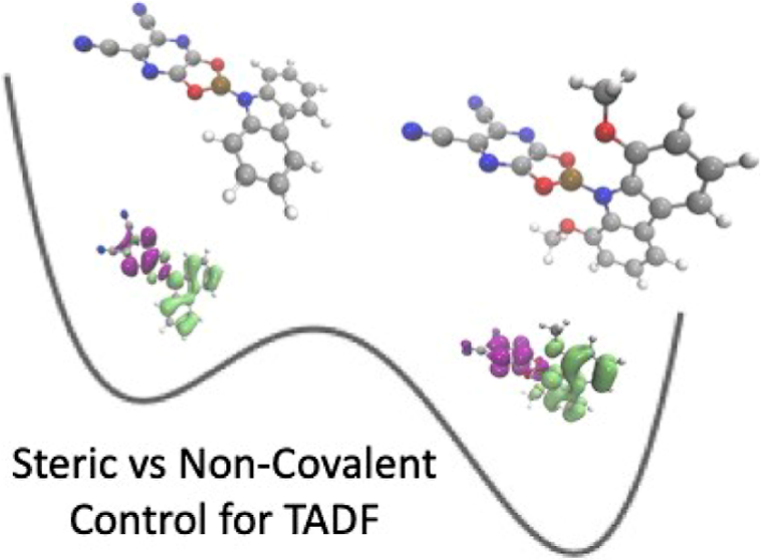

Controlling the architecture of organic molecules is an important aspect in tuning the functional properties of components in organic electronics. For purely organic thermally activated delayed fluorescence (TADF) molecules, design is focused upon orthogonality orientated donor and acceptor units. In these systems, the rotational dynamics around the donor and acceptor bond has been shown to be critical for activating TADF; however, too much conformational freedom can increase the non-radiative rate, leading to a large energy dispersion of the emitting states and conformers, which do not exhibit TADF. To date, control of the motion around the D–A bond has focused upon steric hindrance. In this work, we computationally investigate eight proposed donor–acceptor molecules, exhibiting a B–N bond between the donor and acceptor. We compare the effect of steric hindrance and noncovalent interactions, achieved using oxygen (sulfur) boron heteroatom interactions, in exerting fine conformational control of the excited state dynamics. This work reveals the potential for judiciously chosen noncovalent interactions to strongly influence the functional properties of TADF emitters, including the accessible conformers and the energy dispersion associated with the charge transfer states.

## Introduction

Purely organic compounds exhibiting thermoactively activated delayed fluorescence (TADF) are increasingly being used in a range of applications including organic light emitting diodes (OLEDs),^1,2^ sensors,^3,4^ photocatalysts,^5,6^ imaging,^7,8^ and fluorescence labels.^9,10^ The molecular design of TADF emitters generally requires, with a few exceptions,^11,12^ the use of orthogonality orientated donor (D) and acceptor (A) units.^13,14^ This creates a small spatial overlap between the highest occupied molecular orbital (HOMO) and lowest unoccupied molecular orbital (LUMO), leading to a small energy gap between low lying singlet and triplet states facilitating the use of thermal energy to up-convert nonemitting triplet states into singlet states.^15,16^

While orthogonality between the D and A units is critical, constraining this dihedral bond too rigidly is detrimental to TADF due to a lack of rotational/breathing freedom, which is important for the vibrational coupling mechanism,^17−20^ and tends to yield room temperature phosphorescence instead, i.e., the reversed intersystem crossing (rISC) rate is quenched.^21,22^ Conversely, unconstrained motion around the D–A bond generates a dispersion of TADF rates,^23−25^ broadens the emission width,^26−31^ and leads to an increase in the nonradiative decay rates.^32,33^

Simply incorporating explicit chemical bonds to make the molecules more rigid usually modifies the electronic structure of these emitters too much, preventing TADF, and consequently, other approaches to control the conformation dynamics of donor–acceptor molecules are required. One approach is to introduce steric hindrance between the D–A groups, e.g., methylation;^34−38^ however, this can lead to conformational changes disrupting the orthogonal arrangement between the D and A.^22,39^ An alternative approach is through noncovalent interactions, as their strength should be strong enough to reduce the conformational dynamics without restricting TADF. Toward this goal, Rajamalli and co-workers^40^ proposed an approach based upon hydrogen bonding. Subsequently, Chen et al.,^41^ He et al.,^42^ and Shimoda et al.^43^ invoked D–A hydrogen bonding schemes to develop planar TADF molecules.

However, these derivatives show large (>0.43 eV) Δ*E*_ST_, making TADF at best inefficient. In addition, recent theoretical studies combined with experimental time-resolved spectroscopy studies on several model intramolecular H-bond containing D–A molecules^44,45^ suggested that such D–A hydrogen bonds are unlikely to drive the TADF process. Consequently, the role of intramolecular interactions in controlling the excited state properties of TADF molecules remains an open question.

**Figure 1:**
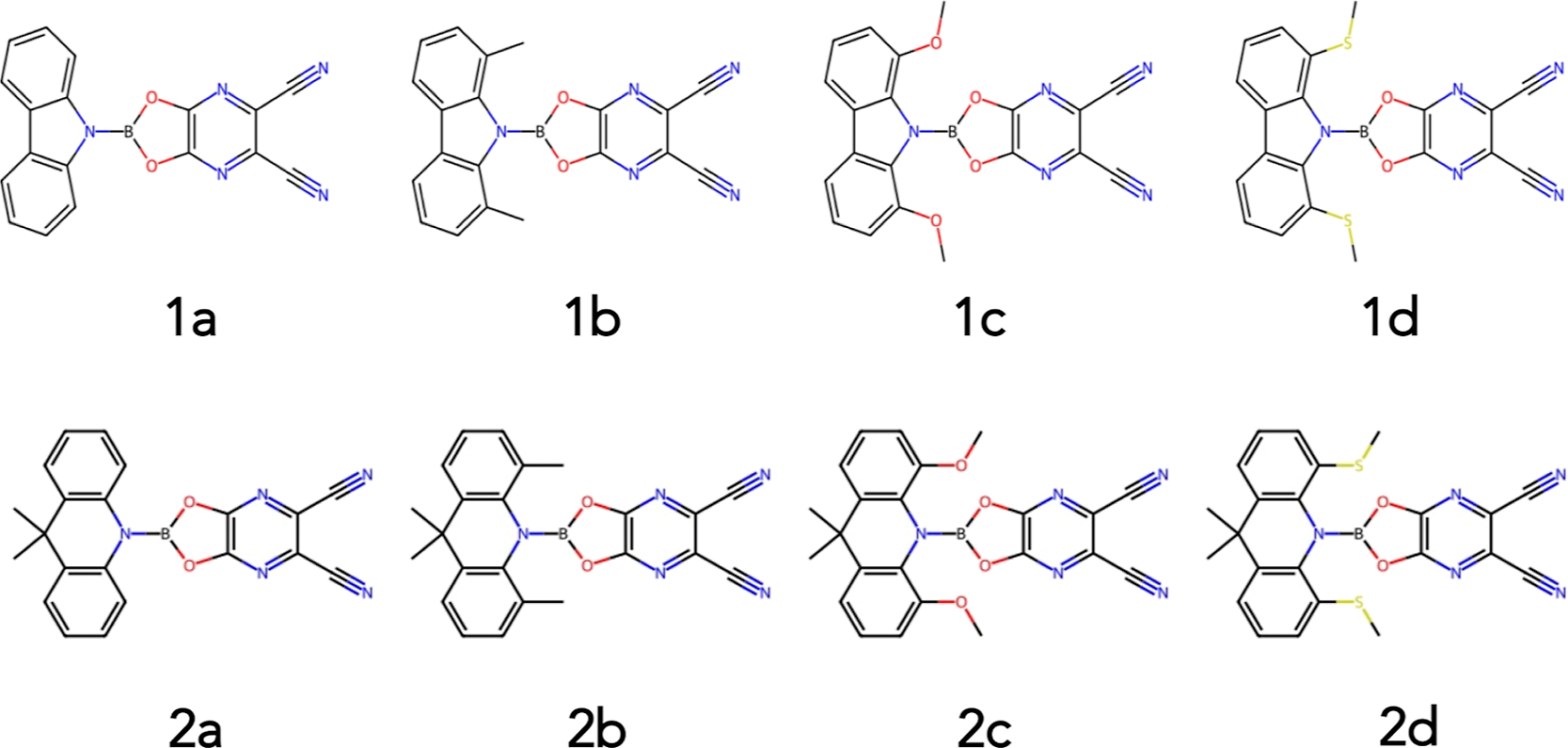
Schematic of the molecular structures considered in this work. The difference between molecules **1** and **2** is a carbozole donor for the former, compared to an acridone donor for the latter.

While the strength of a hydrogen bond is only approximately 5% that of an average covalent bond, incorporating these and similar noncovalent interactions, such as π–π* interactions,^28,46,47^ can have to a significant effect on both electronic ground and excited state properties. Indeed, recently oxygen···boron heteroatom interactions have recently been employed to control the conformation of linear conjugated molecules and polymers.^48,49^ Inspired by these previous works herein, we computationally investigate eight molecules (Figure 1) to compare the effect of steric hindrance and noncovalent interactions (achieved using oxygen···boron and sulfur··boron heteroatom interactions) in exerting fine conformational control of the excited state dynamics of potential TADF emitters. Our molecular design is in part motivated by the work of Wu et al.^50^ and related three-coordinate boron, which have been shown to possess favorable TADF properties.^51−53^ To maintain simplicity, we have limited our study to the D–A framework, i.e., systems which contain only a single D–A bond. Our work, combining quantum chemistry and *ab intio* molecular dynamics, demonstrates the potential for noncovalent interactions to control both the conformational preference of the molecules studied and the dispersion of dihedral angles around the D–A bond. In contrast to steric interactions, the molecules studied in this work exhibiting oxygen···boron noncovalent interactions are locked slightly closer close to orthogonal, a property which promotes favorable TADF characteristics.

## Computational Details

All calculations were performed using the ORCA quantum chemistry software.^54^ Structures were optimized in their electronic ground state and first excited singlet and triplet states using density functional theory (DFT) and linear response time-dependent density functional theory (LR-TDDFT) within the Tamm–Dancoff approximation,^55^ respectively. The LRC-BLYP exchange and correlation functional^56^ was used to address the challenge of charge transfer (CT) excitations.^57^ The optimal tuning (OT) methodology was used to refine the range–separation parameter, ω.^58^ This approach^57,59^ relies on the fact that within the limit of the exact exchange correlation functional, DFT obeys Koopman’s theorem for the energy of the HOMO. Therefore
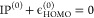
1

Applying the same approach to the anionic system
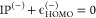
2where ϵ_HOMO_^(−)^ is the energy of the HOMO of the anion and IP^(−)^ is its ionization potential such as
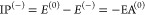
3where EA^(0)^ is the electronic affinity of the neutral form. Equation 2 can therefore be rewritten as
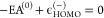
4

The OT approach consists of optimizing the range separation parameter ω, which dictates the switching between the short- and long-range domains of the LRC functional, so that it reproduces the behavior of the exact exchange correlation functional, i.e., minimizing the OT function *J*_OT_^2^(ω)
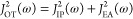
5where
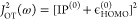
6and
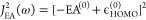
7

Throughout this work, ω_OT_* = 0.2*a*_0_^–1^ was found to be optimal for each molecule, assessed at the electronic ground state geometry. A def2-TZVP basis set^60^ was used, and all energies recorded describe the solvent environment using the conductor-like polarizable continuum medium.^61^ Due to the significant excited state dipole, we note that the linear-response solvation model will not fully stabilize the CT states and therefore is likely to slightly overestimate the emission energies presented herein.^62,63^ To account for weak interactions, central to the present work, Grimme’s D3BJ (D3 with Becke–Johnson damping) dispersion correction method was used in all calculations.^64^ Recent work^65^ has proposed that LC-BLYP functional cannot be reliably combined with optimal-tuning for noncovalent interactions. For the molecules studied in this work, we have found that the functional used does not strongly influence the structures obtained (see potential curves in the Supporting Information) and consequently have retained LC-BLYP, which provides good excitation energies. However, such challenges should be considered and benchmarked when studying these types of system.

Ab initio molecular dynamics (AIMD) were performed in both ground state and first singlet excited state using DFT(PBE0),^66,67^ a def2-SVP basis set,^68^ and Grimme’s D3BJ dispersion correction using the ORCA quantum chemistry software.^54^ Each simulation was initiated from either the ground or first singlet excited state optimized geometry. The temperature was maintained at 300 K for the 20 ps of dynamics. 100 geometries were selected at random from the last 15 ps of the dynamics, and the excited state properties were calculated using the LRC-BLYP exchange and correlation functional, def2-TZVP basis set, and Grimme’s D3BJ dispersion correction, as described above. It is noted that global hybrids are well-known to underestimate the energy of CT states,^69^ and this can lead to D–A geometries, which more strongly tend to a 90° torsion angle to minimize the electron–hole interaction.^70,71^ As shown in the Supporting Information and in agreement with the results presented below, the use of PBE0 during the molecular dynamics, while influencing the total energy of the CT states, does not change the relative energy or stability of the conformers.

**Figure 2:**
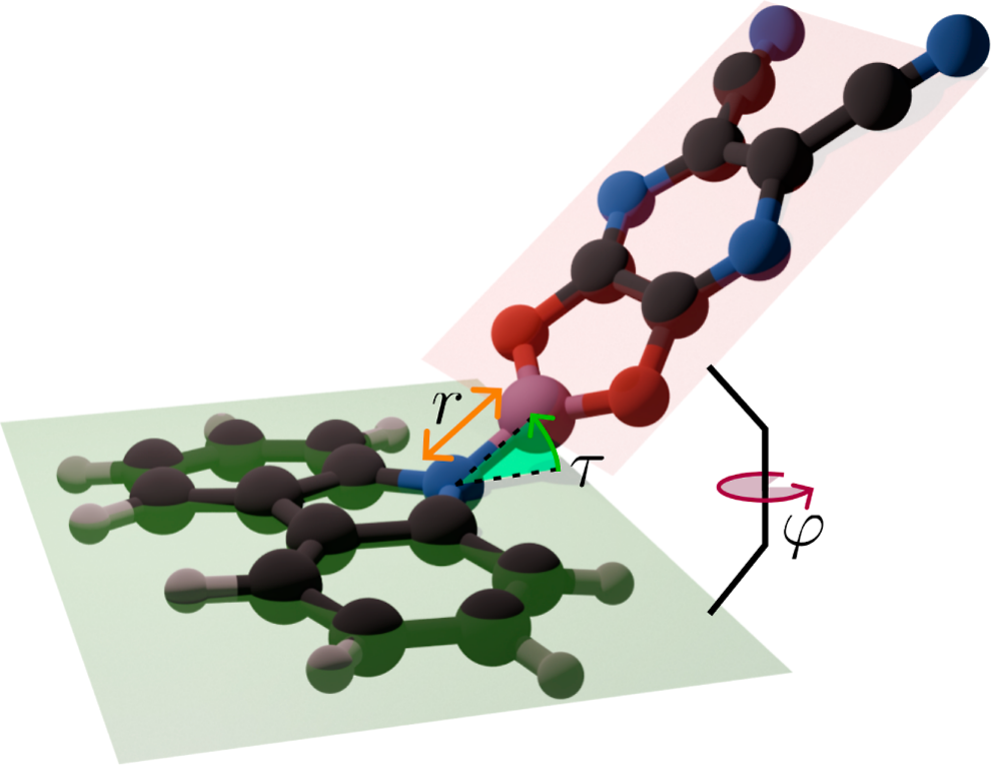
Schematic showing the coordinates used to describe the geometry of the molecules studied in this work. *r* represents the distance between the donor and acceptor, ϕ represents the relative orientation between the D and A, and τ represents the angle between the plane of the D and the plane of the A.

Figure 2 shows a schematic of the structural parameters used to describe the molecular structures throughout this work. *r* represents the distance between the donor and acceptor, ϕ (twist angle) represents the relative orientation between the D and A planes, and τ (bend angle) represents the angle between the plane of the D and the plane of the A. The two angles can be used to define three conformers found in this work: planar (ϕ = 0°, τ = 0°), twisted (ϕ = 90°, τ = 0°), and bent (ϕ = 0°, τ > 15°).

## Results

### Quantum Chemistry Calculations of Critical Points

Tables 1 and 2 show key structural parameters and associated excited state properties of both the carbazole and acridone-based molecules in their energy minimized electronic ground and excited S_1_ and T_1_ states. The full Cartesian coordinates can be found in data repository supporting this publication (see the accessibility statement).

**Table 1:** Key Structural Parameters and Associated Excited State Properties of Molecules **1a**, **1b**, **1c,** and **1d**^a^

	state	conformer	*r*/Å	B-X/Å	∠φ/°	∠_τ_/°	*E*_S1_	*f*_S1_	*E*_T1_	Δ*E*S1–T1	*E*_SOC_
	GS	twisted	1.43		89.2	0.1	3.58	0.00002	3.42	0.16	0.15
	GS	planar*	1.40		0.2	0.1	4.04	0.47984	3.28	0.76	0.05
	T_1_	twisted	1.49		79.2	0.1	3.01	0.01419	3.00	0.01	0.08
**1a**	T_1_	planar*	1.40		0.8	0.5	3.85	0.00007	2.41	1.44	0.38
	S_1_	twisted	1.50		89.7	0.1	2.85	0.00001	2.84	0.01	0.00
	S_1_	planar*	1.49		0.5	0.0	3.11	0.15901	2.86	0.25	0.00
	GS	twisted	1.43	3.00	89.5	0.2	3.44	0.00005	3.40	0.04	0.16
	GS	bent*	1.41	3.31	49.4	37.4	4.05	0.37769	3.27	0.78	0.05
**1b**	T_1_	twisted	1.49	2.95	90.1	0.1	2.67	0.00001	2.67	0.00	0.01
	T_1_	bent*	1.40	3.31	0.0	37.5	3.83	0.01514	2.40	1.43	0.75
	S_1_	twisted*	1.49	3.00	89.7	0.2	2.65	0.00001	2.64	0.01	0.01
	S_1_	bent	1.40	3.32	1.2	35.5	3.25	0.00349	2.59	0.72	1.21
	GS	twisted*	1.44	2.69	89.9	0.0	3.44	0.00007	3.37	0.07	0.15
	GS	bent	1.41	3.08	0.2	31.2	3.77	0.27690	3.25	0.52	0.05
**1c**	T_1_	twisted	1.50	2.73	88.4	0.4	2.50	0.00001	2.50	0.00	0.01
	T_1_	bent*	1.42	2.81	50.8	10.1	3.21	0.03689	2.19	1.02	1.40
	S_1_	twisted*	1.50	2.75	1.2	89.9	2.47	0.00001	2.46	0.01	0.01
	GS	twisted*	1.48	2.83	81.5	0.6	3.62	0.00301	3.15	0.47	0.06
	GS	bent	1.41	3.34	3.22	41.2	3.69	0.14269	3.27	0.42	0.32
**1d**	T_1_	twisted*	1.49	2.90	88.9	5.2	2.45	0.00124	2.44	0.01	0.18
	S_1_	twisted*	1.49	2.91	89.5	6.9	2.37	0.00001	2.35	0.02	0.02

a Only the stable conformers are shown, and the lowest energy conformer for each state is marked with *. The bond distance *r* and angles ϕ and τ are defined in Figure 2. Energies are given in eV and the SOCME are in cm^–1^.

**Table 2:** Key Structural Parameters and Associated Excited State Properties of Molecules **2a**, **2b**, **2c,** and **2d**^a^

	state	conformer	*r*/Å	B-X/Å	∠φ/°	∠_τ_/°	*E*_S1_	*f*_S1_	*E*_T1_	Δ*E*S1–T1	*E*_SOC_
	GS	bent*	1.40		0.1	35.5	4.00	0.44949	3.23	0.77	0.07
	T_1_	twisted	1.50		79.1	0.5	2.41	0.04050	2.36	0.05	0.05
**2a**	T_1_	bent*	1.39		2.3	33.4	3.64	0.10686	2.18	1.36	1.45
	S_1_	twisted*	1.50		89.8	0.0	2.30	0.00001	2.29	0.01	0.02
	S_1_	bent	1.50		1.5	24.2	2.82	0.14981	2.61	0.21	0.14
	GS	bent*	1.39	3.19	0.1	39.9	4.13	0.40272	3.24	0.89	0.05
**2b**	T_1_	bent*	1.39	2.94	2.1	51.6	3.66	0.13260	2.13	1.53	2.54
	S_1_	twisted*	1.51	2.98	81.7	61.2	2.46	0.00421	2.45	0.01	0.11
	S_1_	bent	1.50	3.05	26.2	51.2	2.84	0.11183	2.65	0.19	0.45
	GS	twisted*	1.49	2.15	86.7	24.4	3.65	0.00002	3.12	0.53	0.25
	GS	bent	1.40	2.91	0.2	45.6	3.93	0.23is901	3.22	0.71	0.04
**2c**	T_1_	twisted	1.52	2.30	86.6	0.5	2.24	0.00008	2.23	0.01	0.03
	T_1_	bent*	1.39	2.90	1.3	42.4	3.59	0.07999	2.16	1.43	0.54
	S_1_	twisted*	1.52	2.31	87.7	1.1	2.21	0.00006	2.20	0.01	0.01
	GS	bent*	1.40	3.23	2.1	51.9	3.78	0.01218	3.24	0.54	0.11
**2d**	T_1_	bent*	1.43	3.08	1.0	43.8	2.32	0.00499	2.16	0.16	0.31
	S_1_	bent*	1.47	3.013	32.6	48.34	2.31	0.00308	2.29	0.02	0.31

a Only the stable conformers are shown, and the lowest energy conformer for each state is marked with *. The bond distance *r* and angles φ and τ are defined in Figure 2. Energies are given in eV and the SOCME are in cm^–1^.

In the electronic ground state, **1a** exhibits two conformers, twisted and planar, with the latter being strongly preferred due to it being 0.6 eV lower in energy (Tables S1 and S2). The planar structure exhibits a D–A bond distance of 1.40 Å, which increases to 1.43 Å in the twisted structure due to the reduced overlap between the D and A orbitals arising from orthogonality. Upon excitation into the S_1_ state, the same conformers remain present, but their relative energy gap decreases to 0.18 eV (Tables S1 and S2). This increases the probability to form the twisted conformer, which is more favorable for TADF due to its smaller Δ*E*_S1–T1_ (Table 1), and as shown in Figure S25, there is only a small energy barrier between the two conformers. However, as the planar conformer is dominant in the electronic ground state, this would require a large conformational change, which may be challenging in the solid state media, native for OLED devices.^72^ Both S_1_ optimized structures are accompanied by a ∼0.08 Å elongation of the D–A bond distance, which leads to a large predicted Stokes shift (∼0.7 eV) and a reduction in oscillator strength, spin orbit coupling, and energy gap between the lowest singlet and triplet excited states. In contrast, the lowest triplet state exhibits a single stable planar conformer, associated with a local exciton on the acceptor (Figure S2). Consequently, the molecular structure exhibits comparatively small changes compared to the electronic ground state but a significantly larger Δ*E*_S1–T1_, making TADF impossible.

**Figure 3:**
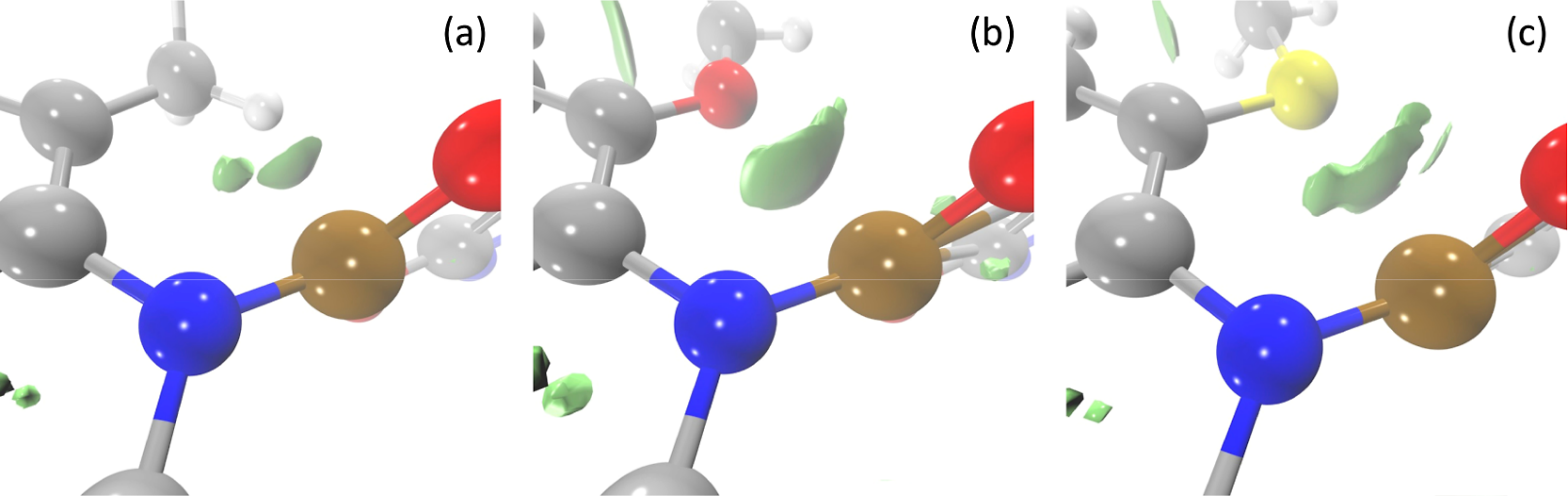
3D isosurface of the reduced density gradient (*s*) isosurface (*s* = 0.3 au) for molecules **1b** (a), **1c** (b), and **1d** (c). All geometries are in the twisted optimized electronic ground state form.

Upon the addition of the methyl (**1b**), methoxy (**1c**), and methylthio (**1d**) groups, the bulky side groups prevent the formation of the planar conformer, meaning that the bent and twisted conformers become dominant. In addition, the relative energy gap between the twisted and bent conformer decreases. For molecule **1b**, this arises from the steric clash between the D and A. In contrast, for methoxy (**1c**) and methylthio (**1d**), this occurs due to the noncovalent bond between the B–N bond and the oxygen (sulfur), arising from the 3-center-2-electron interaction between the oxygen (sulfur) lone pair and low-lying antibonding orbitals of the B–N bond.^73^ Indeed, this electron donation from the lone pair into the B–N bond gives rise to an increase in the D–A bond length in the electronic ground state of the twisted conformers of **1c** and **1d** and makes the twisted conformers of these molecules the lowest energy conformers, even in the electronic ground state. This interaction can be seen using the reduced density gradient (*s*)^75,76^
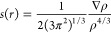
8shown in Figure 3. These highlight stronger contributions between the B–N bond and the oxygen (sulfur) as discussed above.

In the excited S_1_ state, the twisted conformer is the lowest energy conformer for the methyl (**1b**), methoxy (**1c**), and methylthio (**1d**) emitters. This creates the desired orthogonality between the HOMO and LUMO based upon the D and A groups, respectively (Figures S1–S24). With the exception of **1d**, the lowest energy conformer for the excited T_1_ state remains bent and the excited state in this case exhibits a ^3^LE focused upon the A. Importantly, for the methoxy substitute (**1c**) emitter, the energy difference between the twisted and bent conformers in the triplet state is small and the mix of excited state character, i.e., CT and LE is expected to promote TADF.^16^

**Figure 4:**
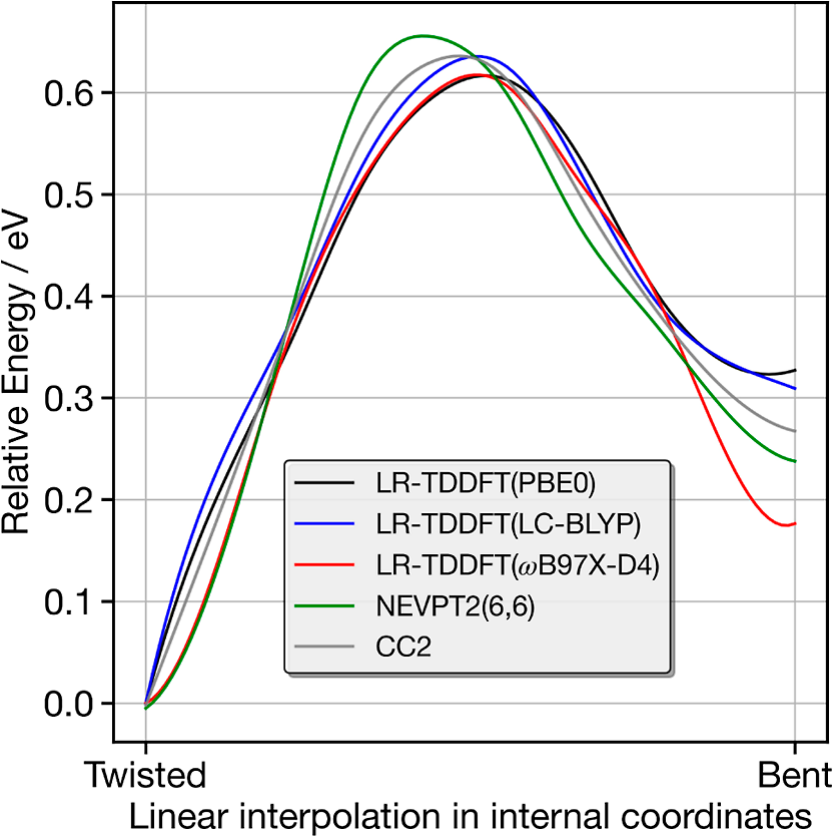
Relative energy of the S_1_ state of **1c** along the LIICs between the twisted and bent ground state optimized structure of **1c**.

The previous section demonstrates the potential for the noncovalent interactions to generate the twisted conformer suitable for TADF. However, the challenge of DFT and LR-TDDFT in describing noncovalent interactions and CT states makes it important to benchmark the accuracy of the potentials, which will influence the relative prevalence of the conformers.^63,77,78^Figure 4 shows the relative energy of the S_1_ state of **1c** along the linear interpolations in internal coordinates (LIICs) between the twisted and bent ground state optimized structures of **1c**. These have been calculated using LR-TDDFT(PBE0), LR-TDDFT(LC-BLYP), LR-TDDFT(ωB97X-D4), NEVPT2(6,6), and CC2. The active space for the NEVPT2 simulations includes the HOMO – 2 to LUMO + 2 orbitals obtained from Hartree–Fock optimization without any additional rotation of the orbitals in the active space. Overall, there is close agreement between the shape of the excited state potential using the different methods (ground and excited state shown in Figure S27), with each showing a preference for the twisted geometry and a high barrier of transformation of just over 0.6 eV. From the LR-TDDFT approaches, ωB97X-D4 exhibits a bent minimum, which is significantly more stable than PBE0 and LC-BLYP; however, the higher level wavefunction methods fall between the two. However, the large size of the barrier between the two minima strongly suggests that once the system is formed within a particular minima, transformation between them will be very slow. For this system, as shown in Figure S27, there is a strong preference for the twisted geometry as the noncovalent interactions lock the D–A in the orthogonal arrangement.

Overall, the simulations of the carbazole emitters demonstrate that the twisted conformers are the only ones likely to exhibit efficient TADF, as both the bent and planar conformers exhibit a larger Δ*E*_S1–T1_. While the steric hindrance of the methyl group somewhat favors the formation of the twisted conformer, it is only upon the inclusion of noncovalent interactions that the structure is sufficiently controlled to promote the twisted conformer in the electronic ground state. This is observed for both the methoxy (**1c**) and methylthio (**1d**) groups, while the latter increases the spin–orbit coupling slightly due to the heavy atom effect.

Table 2 shows the structural parameters and excited state properties of molecules **2a–2d**. In contrast to the carbazole donor, the acridone donor promotes the bent conformer compared to the unsubstituted conformer (**2a**). For both the unsubstituted and methyl substituted molecules, the lowest energy conformer in the electronic ground state is the bent structure, which, owing to the absence of orthogonality between the D and A, gives rise to a large Δ*E*_S1–T1_. Indeed, for the unsubstituted molecule (**2a**), there is no stable twisted conformer, making TADF very unlikely for this particular example. However, as observed for molecules **1a**–**1d**, the addition of the noncovalent interactions via the methoxy group promotes the twisted conformer and exhibits a small Δ*E*_S1–T1_ required for TADF. However, in contrast to the carbozole donor (**1d**), the addition of the methylthio groups (**2d**) leads to only the bent conformer in the electronic ground state, suggesting that only the methoxy substituted systems will be favorable for TADF.

As observed for the carbazole donors, the acridone donor molecules favor the bent conformer in the triplet state, which predominantly exhibits a ^3^LE centered on the A. However, for **2c**, the noncovalent interactions between the B–N and the lone pair oxygen stabilize the twisted conformer, making this comparable (0.1 eV higher in energy, see Tables S13 and S14), which is highly favorable for TADF due to the mixed character of the states. Besides this, the primary difference for the acridone donors is an increase in the excited state energies, associated with the weakening of the donor strength. However, this also generates a larger Stokes shift (∼1.7 eV), which is likely to be problematic in devices, as it will likely to lead to a large radiative rate.

Importantly, these properties are static, i.e., performed at a single optimized geometry. It has been well established that the dynamic properties of molecular emitters^15^ and their conformation are critical in the performance of TADF emitters. Consequently, in the following section, we will combine molecular dynamics with excited state calculations to understand the dynamics.

### Molecular Dynamics of the Conformational Disorder

**Figure 5:**
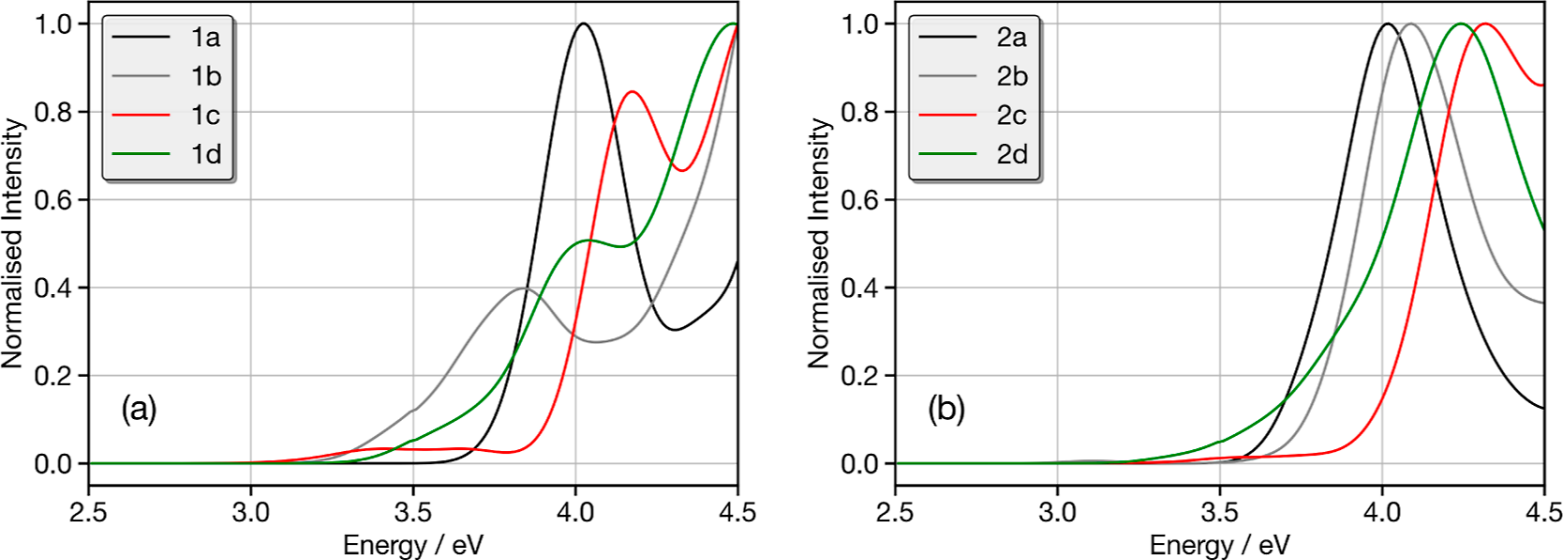
Calculated absorption spectrum of (a) **1a** (black), **1b** (gray), **1c** (red), and **1d** (green) and (b) **2a** (black), **2b** (gray), **2c** (red), and **2d** (green). Each spectrum is calculated by averaging the 100 spectra simulated from structures sampled at random from the ground state AIMD. Each individual spectrum has been broadened using the Gaussian with a full-width at half-maximum of 0.1 eV.

In this section, we seek to understand the influence of the molecular dynamics on the properties of the eight studied emitters, with a particular focus on the influence of the steric (methyl) and noncovalent (methoxy and methylthio) interactions on the structural freedom around the D–A bond. Figure 5 shows the absorption spectrum calculated from 100 snapshots obtained from the ground state AIMD trajectories of molecules **1a**–**1d**; further analysis of these trajectories is shown in the Supporting Information. The absorption spectrum of **1a** shows a single strong band at ∼4.0 eV, associated with transitions into the S_1_ state. As shown in the density differences plot in the Supporting Information (Figure S1), this corresponds to a state which is largely CT in character, but the planar structure leads, as usually expected,^80^ to a stronger overlap between the electron and hole, increasing the oscillator strength and creating a larger (0.90 eV) singlet–triplet splitting.

Upon the addition of the methyl groups (**1b**), the calculated absorption spectrum exhibits three bands below 4.5 eV. The first (∼3.5 eV) is weakest and associated with the orthogonal CT band, with a long tail to lower energy reflecting a dispersion of CT states arising from a large variation and flexibility around the D–A dihedral angle (110 ± 30°, Table S16). The second band (∼3.9 eV) is associated with the S_1_(CT) state in the planar arrangement, with a distorted donor and acceptor, similar to **1a**. The presence of both bands reflects the increased presence of the twisted isomer compared to that of **1a**. The band just visible at 4.5 eV corresponds to transitions into the S_2_ states of both the bent and the twisted isomers.

The addition of the methoxy groups (**1c**), which promote the twisted isomer, significantly changes the shape of the spectrum. The two low lying bands remain, but due to the significantly stronger CT character substantially decreases the oscillator strength of these transitions. This arises due to closer to orthogonal arranges and much smaller dispersion of dihedral angles around the D–A bond (91.6 ± 10°, Table S16). There are two bands above 4.0 eV, which correspond to higher lying CT states and the LE state on the D (Tables S5 and S6).

Finally, the absorption spectrum for **1d** exhibits a similar shape to **1c**. The intensities are slightly modulated arising from the increased dispersion of dihedral distances (Table S16), which permits increased mixing between the D and A, increasing orbital overlap and therefore intensity of the low lying bands. In this case, clearly the 3-center 2-electron interaction between the B–N bond and sulfur lone pair is not as effective at controlling the D–A motion as the methoxy group.

Figure 5b shows the calculated absorption spectrum for **2a**–**2d**. These spectra appear to exhibit much less variation in contrast with Figure 5a. Indeed, the strong band that exists above 4 eV for all molecules arises from transitions into the S_1_ state of the bent conformer, which has a larger oscillator strength due to the orbital overlap. However, as observed for **1c** and **1d**, the absorption spectra of molecules **2c** and **2d** exhibit weak low lying CT bands between 3.5 and 3.8 eV. This is weakest for the methoxy-substituted **2c** as the D–A arrangement is closest to orthogonal and exhibits the smallest dispersion. For **2d**, these low lying CT bands of the twisted conformer gain intensity and appear as long tails on the low energy side of the higher lying band.

Overall, these absorption spectra clearly show that for these molecules, the noncovalent interactions clearly promote the formation of low lying twisted CT bands more than the steric noncovalent interaction. In addition, the methoxy clearly controls the dispersion, giving rise to bands with strongest CT character. It is also importantly to note that the addition of the methoxy and methylthio groups substantially increases the SOC between the lowest lying excited states, which will also promote the ISC/rISC required for TADF.

**Figure 6:**
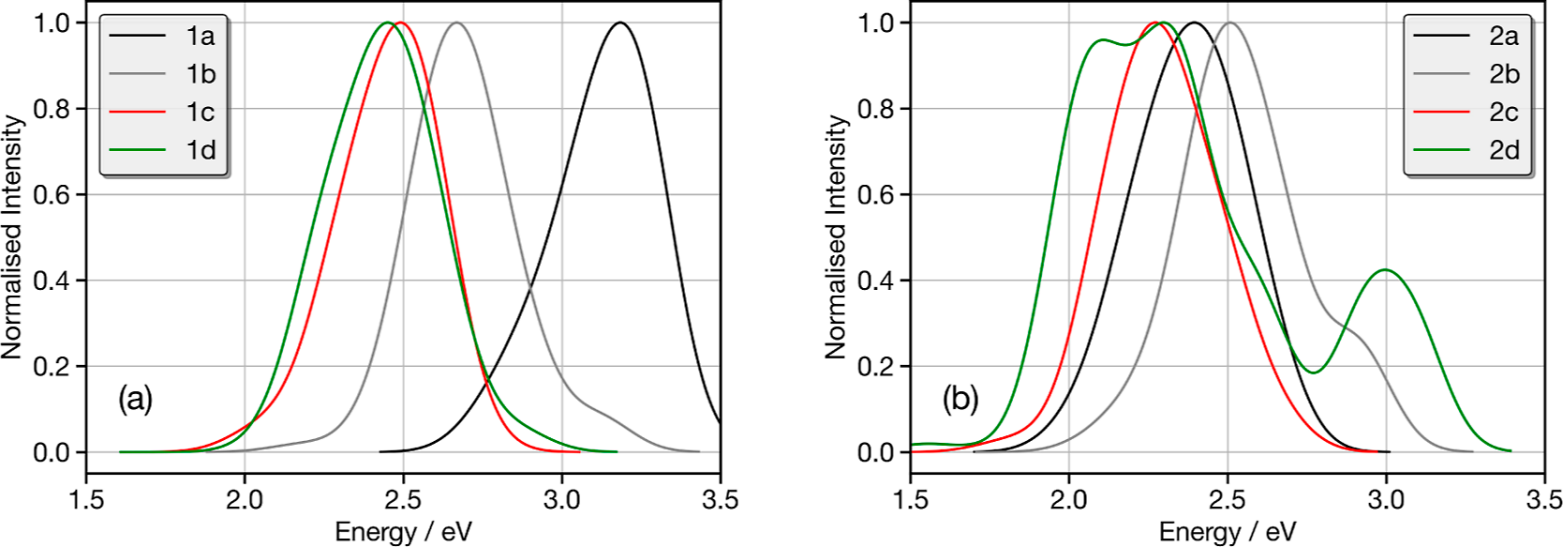
Calculated emission spectrum of (a) **1a** (black), **1b** (gray), **1c** (red), and **1d** (green) and (b) **2a** (black), **2b** (gray), **2c** (red), and **2d** (green). Each spectrum is calculated by averaging the 100 spectra simulated from structures sampled at random from the excited S_1_ AIMD. Each individual spectrum has been broadened using the Gaussian with a full-width at half-maximum of 0.1 eV.

Figure 6a shows the emission spectra of molecules **1a**–**1d** calculated from the S_1_ excited state AIMD trajectories. Molecule **1a** shows a band centered at 3.1 eV, which corresponds to emission from the low lying CT state associated with the planar structure. This band exhibits a distinct asymmetry (∼2.6 eV) that arises from a weaker contribution of the twisted conformers. Upon the addition of the methyl groups, the two same bands (∼2.6 and 3.1 eV) remain present, but the relative intensity of the two bands is completely switched due to the increased prevalence of the twisted conformer in the excited state. The emission for **1c** and **1d** shows a single broad band in both cases centered just below 2.5 eV. This arises from emission from the lowest singlet excited state of the twisted conformer, which is dominant in these noncovalently controlled structures.

The emission spectra for molecules **2a–2d** exhibit quite a different trend. **2a** exhibits a single band at ∼2.3 eV, which corresponds to the bent conformer that dominates the AIMD. The addition of the methyl groups (**2b**) generates two bands at 2.5 and 2.9 eV arising from the twisted and bent conformers, which both appear for this system. A similar structure for emission is also observed for **2d,** which also exhibits both conformers. Finally, the emission of **2c** exhibits a single band, associated with the twisted conformer, which is preferred due to the noncovalent interactions.

Overall, the emission properties of compounds **1c** and **2c** are clearly preferred. The emission is both the narrowest and the strong CT character favorable for TADF. We note that the emission spectra in Figure 6 have all been normalized to the largest intensity peak. Figure S16 shows the average oscillator strength, and as expected, this is weakest for molecules **1c** and **2c** due to the strong CT character.

## Discussion and Conclusions

In this article, we have investigated a series of donor–acceptor molecules, exhibiting a B–N bond between the donor and acceptor with the objective of understanding the interplay of steric hindrance and noncovalent interactions in exerting fine conformational control on the excited state properties. Our results show that for these molecules, there are three major conformers, namely, planar, bent, and twisted, which differ in the structure around the D–A bond. In the electronic ground state, both the unhindered (**1a** and **2a**) and sterically methyl hindered (**1b** and **2b**) molecules favor the planar or bent conformers. This increases the communication between the D and A and therefore increases the energy gap between the singlet and triplet states, making TADF unfavorable. While the electronically excited states decrease the energy gap between the planar/bent and twisted conformers, generating the favorable twisted conformer in the excited state will require a significant structural rearrangements. While such changes are possible in the unconstrained environment of solution, the probability of it occurring is in the restricted solid state medium of an OLED.

The addition of noncovalent interactions (**1c**, **1d**, **2c,** and **2d**) arising from the 3-center-2-electron interaction between the oxygen (sulfur) lone pair and low-lying antibonding orbitals of the B–N bond gives rise to a somewhat difference picture. Indeed, these interactions strongly stabilize the twisted conformer required for these emitters to exhibit favorable TADF properties. In addition, the presence of oxygen and sulfur increases the spin orbit coupling, increasing the rate of ISC/rISC.

Overall, the molecules presented herein represent one of the first molecular frameworks, making it possible to fairly assess the influence of steric and noncovalent interactions in dictating the conformation and functional properties of TADF molecules, i.e., without substantially changing the geometry and electronic structure. While hydrogen bonding previously proposed^40^ to contribute in the interaction of TADF is a stronger interaction than the noncovalent interactions used herein, we provide unambiguous evidence that through the careful design of the molecular structure, noncovalent interactions are able to effectively control the conformation of TADF molecules, offering new design directions of TADF emitters. This class of noncovalent interaction used for TADF emitters not only offers the opportunity to fine tune excited state functional properties such as energy gap and emission energy but also control important dynamic properties which are detrimental to TADF materials. Indeed, unconstrained D–A molecules tend to exhibit CT emission, which dynamically shifts in time arising from energy dispersion cause by dihedral angle inhomogeneity in the most twisted D–A molecule.^81^ The narrowing of the distribution around the D–A bond, especially for the methoxy derivatives arising from the noncovalent interactions, offers the opportunity to remove this dynamical dispersive behavior and ultimately narrow the emission achieving molecules more suitable for OLED application.

In this computational study, we have not focused upon the synthetic ability of the proposed species, but rather the impact of the steric and noncovalent interactions. Further work should translate these concepts into molecules which are readily synthesized and measured spectroscopically. Indeed, the core ingredient in this case will be the B–N bond, which allows for the introduction of the noncovalent interactions via the methoxy without disruption of the orthogonal arrangement. While other noncovalent interactions, such as hydrogen bonds, have been integrated into TADF molecules, the requirements of hydrogen bonds significantly distort the structures, making a comparison between different systems challenging.

## Data Availability

The data supporting this publication are openly available under an Open Data Commons Open Database License. Additional metadata are available at 10.25405/data.ncl.25962481.

## Supporting Information Available

Density different plots of the excited state for each molecule, potential energy curves along reaction coordinations between conformers, and tables of optimized structure and excited state energetics and character (PDF)
