# Supplementary material for: Conformational Control of Donor–Acceptor Molecules Using Non-covalent Interactions
Source: J Phys Chem A. 2024 Sep 17;128(38):8035–44. doi: 10.1021/acs.jpca.4c03711 (PMC11440601; doi:10.1021/acs.jpca.4c03711)
Supplement: Supplementary file 1 — jp4c03711_si_001.pdf [file jp4c03711_si_001.pdf]

Supporting Information: Conformational Control of Donor-Acceptor Molecules using Non-Covalent Interactions

Shawana Ahmad, Julien Eng, and Thomas J. Penfold*

Chemistry - School of Natural and Environmental Sciences, Newcastle University, Newcastle upon-Tyne, NE1 7RU, United Kingdom

E-mail: tom.penfold@ncl.ac.uk

List of Figures

S1 Structure and S_1 density difference associated with the ground state optimised structure of **1a**. S16

S2 Structure and T_1 density difference associated with the T_1 state optimised structure of **1a**. S16

S3 Structure and S_1 density difference associated with the S_1 state optimised structure of **1a**. S17

S4 Structure and S_1 density difference associated with the ground state optimised structure of **1b**. S17

S5 Structure and T_1 density difference associated with the T_1 state optimised structure of **1b**. S18

S6 Structure and S_1 density difference associated with the S_1 state optimised structure of **1b**. S18

S7 Structure and S_1 density difference associated with the ground state optimised structure of **1c**. S19
S8 Structure and T_1 density difference associated with the T_1 state optimised structure of **1c**. S19
S9 Structure and S_1 density difference associated with the S_1 state optimised structure of **1c**. S20
S10 Structure and S_1 density difference associated with the ground state optimised structure of **1d**. S20
S11 Structure and T_1 density difference associated with the T_1 state optimised structure of **1d**. S21
S12 Structure and S_1 density difference associated with the S_1 state optimised structure of **1d**. S21
S13 Structure and S_1 density difference associated with the ground state optimised structure of **2a**. S22
S14 Structure and T_1 density difference associated with the T_1 state optimised structure of **2a**. S22
S15 Structure and S_1 density difference associated with the S_1 state optimised structure of **2a**. S23
S16 Structure and S_1 density difference associated with the ground state optimised structure of **2b**. S23
S17 Structure and T_1 density difference associated with the T_1 state optimised structure of **2b**. S24
S18 Structure and S_1 density difference associated with the S_1 state optimised structure of **2b**. S24
S19 Structure and S_1 density difference associated with the ground state optimised structure of **2c**. S25

S20 Structure and T_1 density difference associated with the T_1 state optimised structure of **2c**. S25
S21 Structure and S_1 density difference associated with the S_1 state optimised structure of **2c**. S26
S22 Structure and S_1 density difference associated with the ground state optimised structure of **2d**. S26
S23 Structure and T_1 density difference associated with the T_1 state optimised structure of **2d**. S27
S24 Structure and S_1 density difference associated with the S_1 state optimised structure of **2d**. S27
S25 Potential of the ground and excited state along a linear interpolations in internal coordinates (LIICs) between the twisted and planar ground state optimised structures of **1a**. Blue is calculated using the PBE0 functional, black with the optimally tuned LC-BLYP and red with the optimally tuned ωB97X-D4 exchange and correlation functional. S28
S26 Potential of the ground and excited state along a linear interpolations in internal coordinates (LIICs) between the twisted and bent ground state optimised structures of **1b**. Blue is calculated using the PBE0 functional, black with the optimally tuned LC-BLYP and red with the optimally tuned ωB97X-D4 exchange and correlation functional. S29
S27 Potential of the ground and excited state along a linear interpolations in internal coordinates (LIICs) between the twisted and bent ground state optimised structures of **1c**. Blue is calculated using the PBE0 functional, black with the optimally tuned LC-BLYP and red with the optimally tuned ωB97X-D4 exchange and correlation functional. S29

S28 Potential of the ground and excited state along a linear interpolations in internal coordinates (LIICs) between the twisted and bent ground state optimised structures of **1d**. Blue is calculated using the PBE0 functional, black with the optimally tuned LC-BLYP and red with the optimally tuned ωB97X-D4 exchange and correlation functional. S30

List of Tables

S1 Electronic structure at the optimised geometry of the electronic ground and lowest S_1 and T_1 states of the twisted conformer of **1a**. All energies relative to the lowest energy conformer of the **1a** molecule (planar). CT = charge transfer, LE(A) = Local exciton on the acceptor and LE(D) = Local exciton on the donor. S8

S2 Electronic structure at the optimised geometry of the electronic ground and lowest S_1 and T_1 states of the planar conformer of **1a**. All energies relative to the lowest energy conformer of the **1a** molecule (planar). CT = charge transfer, LE(A) = Local exciton on the acceptor and LE(D) = Local exciton on the donor. S8

S3 Electronic structure at the optimised geometry of the electronic ground and lowest S_1 and T_1 states of the twisted conformer of **1b**. All energies relative to the lowest energy conformer of the **1b** molecule (bent). CT = charge transfer, LE(A) = Local exciton on the acceptor and LE(D) = Local exciton on the donor. S9

S4 Electronic structure at the optimised geometry of the electronic ground and lowest S_1 and T_1 states of the bent conformer of **1b**. All energies relative to the lowest energy conformer of the **1b** molecule (bent). CT = charge transfer, LE(A) = Local exciton on the acceptor and LE(D) = Local exciton on the donor. S9
S5 Electronic structure at the optimised geometry of the electronic ground and lowest S_1 and T_1 states of the twisted conformer of **1c**. All energies relative to the lowest energy conformer of the **1c** molecule (twisted). CT = charge transfer, LE(A) = Local exciton on the acceptor and LE(D) = Local exciton on the donor. S10
S6 Electronic structure at the optimised geometry of the electronic ground and lowest S_1 and T_1 states of the bent conformer of **1c**. All energies relative to the lowest energy conformer of the **1c** molecule (twisted). CT = charge transfer, LE(A) = Local exciton on the acceptor and LE(D) = Local exciton on the donor. S10
S7 Electronic structure at the optimised geometry of the electronic ground and lowest S_1 and T_1 states of the twisted conformer of **1d**. All energies relative to the lowest energy conformer of the **1d** molecule (twisted). CT = charge transfer, LE(A) = Local exciton on the acceptor and LE(D) = Local exciton on the donor. S11
S8 Electronic structure at the optimised geometry of the electronic ground and lowest S_1 and T_1 states of the bent conformer of **1d**. All energies relative to the lowest energy conformer of the **1d** molecule (twisted). CT = charge transfer, LE(A) = Local exciton on the acceptor and LE(D) = Local exciton on the donor. S11

S9 Electronic structure at the optimised geometry of the electronic ground and lowest S_1 and T_1 states of the twisted conformer of **2a**. All energies relative to the lowest energy conformer of the **2a** molecule (bent). CT = charge transfer, LE(A) = Local exciton on the acceptor and LE(D) = Local exciton on the donor. S12
S10 Electronic structure at the optimised geometry of the electronic ground and lowest S_1 and T_1 states of the bent conformer of **2a**. All energies relative to the lowest energy conformer of the **2a** molecule (bent). CT = charge transfer, LE(A) = Local exciton on the acceptor and LE(D) = Local exciton on the donor. S12
S11 Electronic structure at the optimised geometry of the electronic ground and lowest S_1 and T_1 states of the twisted conformer of **2b**. All energies relative to the lowest energy conformer of the **2b** molecule (bent). CT = charge transfer, LE(A) = Local exciton on the acceptor and LE(D) = Local exciton on the donor. S13
S12 Electronic structure at the optimised geometry of the electronic ground and lowest S_1 and T_1 states of the bent conformer of **2b**. All energies relative to the lowest energy conformer of the **2b** molecule (bent). CT = charge transfer, LE(A) = Local exciton on the acceptor and LE(D) = Local exciton on the donor. S13
S13 Electronic structure at the optimised geometry of the electronic ground and lowest S_1 and T_1 states of the twisted conformer of **2c**. All energies relative to the lowest energy conformer of the **2c** molecule (twisted). CT = charge transfer, LE(A) = Local exciton on the acceptor and LE(D) = Local exciton on the donor. S14

S14 Electronic structure at the optimised geometry of the electronic ground and lowest S_1 and T_1 states of the bent conformer of **2c**. All energies relative to the lowest energy conformer of the **2c** molecule (twisted). CT = charge transfer, LE(A) = Local exciton on the acceptor and LE(D) = Local exciton on the donor. S14
S15 Electronic structure at the optimised geometry of the electronic ground and lowest S_1 and T_1 states of the bent conformer of **2d**. All energies relative to the lowest energy conformer of the **2d** molecule (twisted). CT = charge transfer, LE(A) = Local exciton on the acceptor and LE(D) = Local exciton on the donor. S15
S16 Analysis of the geometric and electronic structure properties obtained from 15 ps of *ab initio* molecular dynamics in the electronic ground (S_0) and first singlet excited state (S_1). S31

Table S1: Electronic structure at the optimised geometry of the electronic ground and lowest S_1 and T_1 states of the twisted conformer of **1a**. All energies relative to the lowest energy conformer of the **1a** molecule (planar). CT = charge transfer, LE(A) = Local exciton on the acceptor and LE(D) = Local exciton on the donor.

Ground State			T_1^{Min}		S_1^{Min}	
State	Nature	ΔE / eV	Nature	ΔE / eV	Nature	ΔE / eV
S_0	–	0.56	–	0.86	–	1.03
S_1	CT	4.13	CT	3.87	CT	3.88
S_2	CT	4.64	CT	4.56	CT	4.61
S_3	CT	4.74	CT	4.71	CT	4.79
T_1	LE(A)	3.98	CT	3.86	CT	3.87
T_2	LE(A)	4.09	LE(A)	4.19	LE(A)	4.29
T_3	CT	4.12	LE(D)	4.35	LE(D)	4.50

Table S2: Electronic structure at the optimised geometry of the electronic ground and lowest S_1 and T_1 states of the planar conformer of **1a**. All energies relative to the lowest energy conformer of the **1a** molecule (planar). CT = charge transfer, LE(A) = Local exciton on the acceptor and LE(D) = Local exciton on the donor.

Ground State			T_1^{Min}		S_1^{Min}	
State	Nature	ΔE / eV	Nature	ΔE / eV	Nature	ΔE / eV
S_0	–	0.00	–	0.44	–	0.59
S_1	CT + LE(A)	4.04	LE(A)	4.29	CT	3.70
S_2	CT	4.40	CT	4.36	CT	4.28
S_3	CT	4.44	CT + LE(A)	4.51	CT	4.55
T_1	LE(A)	3.28	LE(A)	2.85	CT + LE(A)	3.44
T_2	LE(A) + CT	3.37	LE(A)	3.77	LE(A) + CT	4.02
T_3	LE(D)	3.59	LE(D)	4.03	LE(A) + CT	4.15

Table S3: Electronic structure at the optimised geometry of the electronic ground and lowest S_1 and T_1 states of the twisted conformer of **1b**. All energies relative to the lowest energy conformer of the **1b** molecule (bent). CT = charge transfer, LE(A) = Local exciton on the acceptor and LE(D) = Local exciton on the donor.

Ground State			T_1^{Min}		S_1^{Min}	
State	Nature	ΔE / eV	Nature	ΔE / eV	Nature	ΔE / eV
S_0	–	0.18	–	0.64	–	0.67
S_1	CT	3.62	CT	3.31	CT	3.32
S_2	CT	4.19	CT	4.15	CT	4.15
S_3	CT	4.20	CT	4.21	CT	4.23
T_1	LE(A)	3.58	CT	3.31	CT	3.31
T_2	CT + LE(D)	3.61	LE(A)	3.89	LE(A)	3.91
T_3	LE(A)	3.68	LE(D)	4.05	LE(D)	4.08

Table S4: Electronic structure at the optimised geometry of the electronic ground and lowest S_1 and T_1 states of the bent conformer of **1b**. All energies relative to the lowest energy conformer of the **1b** molecule (bent). CT = charge transfer, LE(A) = Local exciton on the acceptor and LE(D) = Local exciton on the donor.

Ground State			T_1^{Min}		S_1^{Min}	
State	Nature	ΔE / eV	Nature	ΔE / eV	Nature	ΔE / eV
S_0	–	0.00	–	0.45	–	0.66
S_1	CT	4.05	LE(A)	4.28	LE(A)	3.91
S_2	CT	4.35	CT	4.35	CT	4.61
S_3	CT	4.42	CT + LE(A)	4.53	CT	4.87
T_1	LE(A)	3.27	LE(A)	2.85	LE(A)	3.19
T_2	LE(A)	3.38	LE(A)	3.74	LE(A)	3.84
T_3	LE(D)	3.84	LE(D)	3.94	LE(D)	4.51

Table S5: Electronic structure at the optimised geometry of the electronic ground and lowest S_1 and T_1 states of the twisted conformer of **1c**. All energies relative to the lowest energy conformer of the **1c** molecule (twisted). CT = charge transfer, LE(A) = Local exciton on the acceptor and LE(D) = Local exciton on the donor.

Ground State			T_1^{Min}		S_1^{Min}	
State	Nature	ΔE / eV	Nature	ΔE / eV	Nature	ΔE / eV
S_0	–	0.00	–	0.53	–	0.56
S_1	CT	3.44	CT	3.03	CT	3.03
S_2	CT	3.89	CT	3.89	CT	3.90
S_3	LE(D)	4.30	CT	4.09	CT	4.09
T_1	LE(A)	3.37	CT	3.03	CT	3.02
T_2	CT	3.43	LE(A)	3.75	LE(A)	3.78
T_3	LE(A)	3.51	LE(D)	3.85	LE(D)	3.88

Table S6: Electronic structure at the optimised geometry of the electronic ground and lowest S_1 and T_1 states of the bent conformer of **1c**. All energies relative to the lowest energy conformer of the **1c** molecule (twisted). CT = charge transfer, LE(A) = Local exciton on the acceptor and LE(D) = Local exciton on the donor.

Ground State			T_1^{Min}		S_1^{Min}	
State	Nature	ΔE / eV	Nature	ΔE / eV	Nature	ΔE / eV
S_0	–	0.12	–	0.72	–	–
S_1	CT	3.89	CT	4.04	–	–
S_2	CT	4.25	CT + LE(A)	4.37	–	–
S_3	LE(D)	4.47	LE(A)	4.46	–	–
T_1	LE(A)	3.37	LE(A)	2.91	–	–
T_2	LE(A) + CT	3.44	LE(A)	3.87	–	–
T_3	LE(D)	3.73	CT	4.09	–	–

Table S7: Electronic structure at the optimised geometry of the electronic ground and lowest S_1 and T_1 states of the twisted conformer of **1d**. All energies relative to the lowest energy conformer of the **1d** molecule (twisted). CT = charge transfer, LE(A) = Local exciton on the acceptor and LE(D) = Local exciton on the donor.

Ground State			T_1^{Min}		S_1^{Min}	
State	Nature	ΔE / eV	Nature	ΔE / eV	Nature	ΔE / eV
S_0	–	0.00	–	0.29	–	0.37
S_1	CT	3.62	CT	2.74	CT	2.74
S_2	CT + LE(D)	3.88	CT	3.34	CT	3.39
S_3	CT	3.90	CT	3.65	CT	3.67
T_1	LE(A)	3.15	CT	2.73	CT	2.72
T_2	LE(D)	3.22	CT	3.31	CT	3.37
T_3	LE(A)	3.32	LE(D)	3.49	LE(D)	3.56

Table S8: Electronic structure at the optimised geometry of the electronic ground and lowest S_1 and T_1 states of the bent conformer of **1d**. All energies relative to the lowest energy conformer of the **1d** molecule (twisted). CT = charge transfer, LE(A) = Local exciton on the acceptor and LE(D) = Local exciton on the donor.

Ground State			T_1^{Min}		S_1^{Min}	
State	Nature	ΔE / eV	Nature	ΔE / eV	Nature	ΔE / eV
S_0	–	0.42	–	–	–	–
S_1	CT	4.11	–	–	–	–
S_2	CT	4.30	–	–	–	–
S_3	CT	4.44	–	–	–	–
T_1	LE(A)	3.69	–	–	–	–
T_2	LE(D)	3.76	–	–	–	–
T_3	LE(A) + CT	3.77	–	–	–	–

Table S9: Electronic structure at the optimised geometry of the electronic ground and lowest S_1 and T_1 states of the twisted conformer of **2a**. All energies relative to the lowest energy conformer of the **2a** molecule (bent). CT = charge transfer, LE(A) = Local exciton on the acceptor and LE(D) = Local exciton on the donor.

Ground State			T_1^{Min}		S_1^{Min}	
State	Nature	ΔE / eV	Nature	ΔE / eV	Nature	ΔE / eV
S_0	–	–	–	0.63	–	0.71
S_1	–	–	CT	3.04	CT	3.01
S_2	–	–	CT	3.91	CT	3.91
S_3	–	–	LE(D)	4.65	LE(D)	4.88
T_1	–	–	CT	2.99	CT	3.00
T_2	–	–	CT	3.85	CT	3.91
T_3	–	–	LE(A)	3.92	LE(A)	3.96

Table S10: Electronic structure at the optimised geometry of the electronic ground and lowest S_1 and T_1 states of the bent conformer of **2a**. All energies relative to the lowest energy conformer of the **2a** molecule (bent). CT = charge transfer, LE(A) = Local exciton on the acceptor and LE(D) = Local exciton on the donor.

Ground State			T_1^{Min}		S_1^{Min}	
State	Nature	ΔE / eV	Nature	ΔE / eV	Nature	ΔE / eV
S_0	–	0.00	–	0.30	–	0.39
S_1	CT	4.01	LE(A) + CT	3.94	CT	3.21
S_2	CT	4.33	LE(A)	4.10	CT	4.03
S_3	LE(A)	4.51	LE(A) + CT	4.25	LE(A)	4.73
T_1	LE(A)	3.23	LE(A)	2.48	CT	3.00
T_2	LE(A) + CT	3.32	LE(A)	3.44	LE(A) + CT	3.70
T_3	LE(A)	3.95	LE(A)	4.02	LE(A)	3.79

Table S11: Electronic structure at the optimised geometry of the electronic ground and lowest S_1 and T_1 states of the twisted conformer of **2b**. All energies relative to the lowest energy conformer of the **2b** molecule (bent). CT = charge transfer, LE(A) = Local exciton on the acceptor and LE(D) = Local exciton on the donor.

Ground State			T_1^{MIN}		S_1^{MIN}	
State	Nature	ΔE / eV	Nature	ΔE / eV	Nature	ΔE / eV
S_0	–	–	–	–	–	1.18
S_1	–	–	–	–	CT	3.64
S_2	–	–	–	–	CT	4.56
S_3	–	–	–	–	CT	5.25
T_1	–	–	–	–	CT	3.63
T_2	–	–	–	–	LE(A)	4.41
T_3	–	–	–	–	CT	4.54

Table S12: Electronic structure at the optimised geometry of the electronic ground and lowest S_1 and T_1 states of the bent conformer of **2b**. All energies relative to the lowest energy conformer of the **2b** molecule (bent). CT = charge transfer, LE(A) = Local exciton on the acceptor and LE(D) = Local exciton on the donor.

Ground State			T_1^{MIN}		S_1^{MIN}	
State	Nature	ΔE / eV	Nature	ΔE / eV	Nature	ΔE / eV
S_0	–	0.00	–	0.70	–	0.83
S_1	CT + LE(A)	4.14	CT	4.46	CT	3.67
S_2	CT + LE(A)	4.47	CT	4.48	CT	4.50
S_3	LE(A)	4.50	CT	4.71	CT	4.96
T_1	LE(A)	3.24	CT	2.73	CT	3.48
T_2	LE(A)	3.63	LE(A)	3.84	CT + LE(A)	4.17
T_3	LE(A)	3.97	LE(A)	4.29	LE(A)	4.20

Table S13: Electronic structure at the optimised geometry of the electronic ground and lowest S_1 and T_1 states of the twisted conformer of **2c**. All energies relative to the lowest energy conformer of the **2c** molecule (twisted). CT = charge transfer, LE(A) = Local exciton on the acceptor and LE(D) = Local exciton on the donor.

Ground State			T_1^{MIN}		S_1^{MIN}	
State	Nature	ΔE / eV	Nature	ΔE / eV	Nature	ΔE / eV
S_0	–	0.00	–	0.85	–	0.88
S_1	CT	3.65	CT	3.25	CT	3.09
S_2	CT	3.82	CT	3.94	CT	3.95
S_3	LE(D)	4.25	CT	4.41	CT	4.41
T_1	LE(A)	3.12	CT	3.08	CT	3.08
T_2	LE(A)	3.38	CT	3.93	CT	3.94
T_3	LE(D)	3.45	LE(A)	3.95	LE(A)	4.68

Table S14: Electronic structure at the optimised geometry of the electronic ground and lowest S_1 and T_1 states of the bent conformer of **2c**. All energies relative to the lowest energy conformer of the **2c** molecule (twisted). CT = charge transfer, LE(A) = Local exciton on the acceptor and LE(D) = Local exciton on the donor.

Ground State			T_1^{MIN}		S_1^{MIN}	
State	Nature	ΔE / eV	Nature	ΔE / eV	Nature	ΔE / eV
S_0	–	0.15	–	0.80	–	–
S_1	CT	4.08	CT	4.39	–	–
S_2	CT	4.39	LE(A)	4.54	–	–
S_3	LE(A)	4.63	LE(A) + CT	4.75	–	–
T_1	LE(A)	3.37	LE(A)	2.96	–	–
T_2	LE(A)	3.48	LE(A)	3.95	–	–
T_3	LE(D)	4.04	LE(A)	4.40	–	–

Table S15: Electronic structure at the optimised geometry of the electronic ground and lowest S_1 and T_1 states of the bent conformer of **2d**. All energies relative to the lowest energy conformer of the **2d** molecule (twisted). CT = charge transfer, LE(A) = Local exciton on the acceptor and LE(D) = Local exciton on the donor.

Ground State			T_1^{MIN}		S_1^{MIN}	
State	Nature	ΔE / eV	Nature	ΔE / eV	Nature	ΔE / eV
S_0	–	0.00	–	1.08	–	0.92
S_1	CT	3.78	CT	3.30	CT	3.24
S_2	CT	3.99	CT	4.21	CT	4.08
S_3	CT	4.09	CT	4.25	CT	4.14
T_1	LE(A)	3.24	CT	3.22	CT	3.22
T_2	LE(A)	3.39	CT + LE(A)	3.99	LE(A)	3.92
T_3	CT + LE(D)	3.70	CT	4.11	LE(D)	4.04

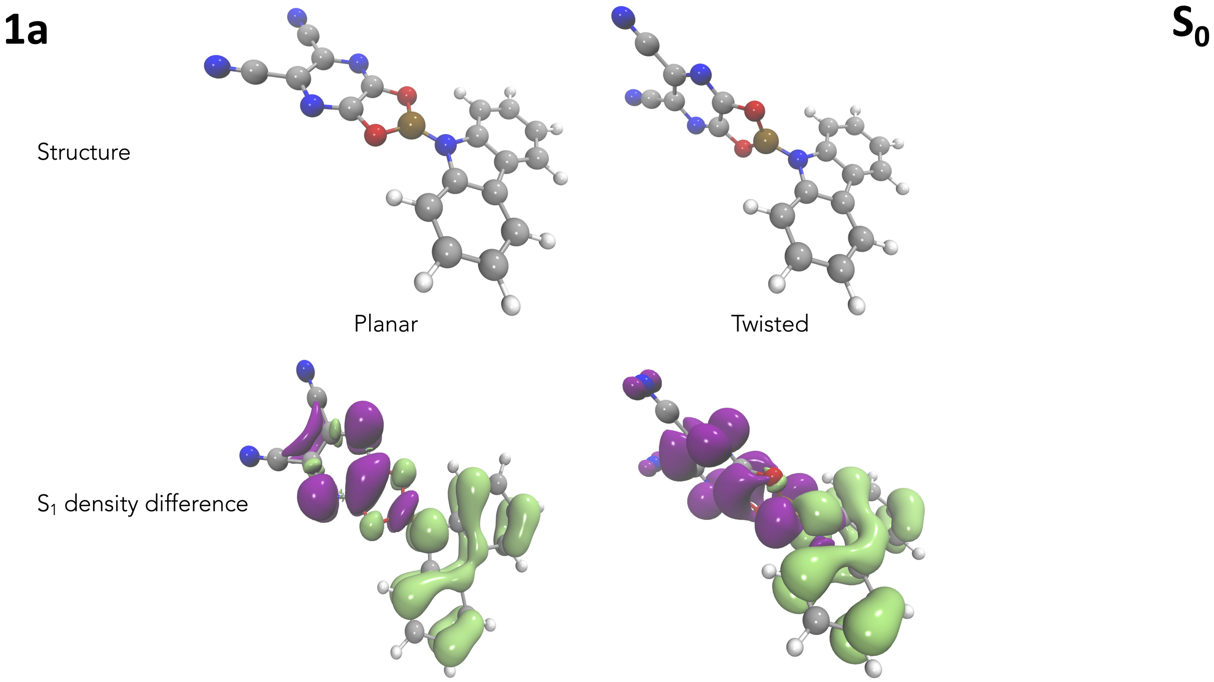

Figure S1: Structure and S_1 density difference associated with the ground state optimised structure of **1a**.

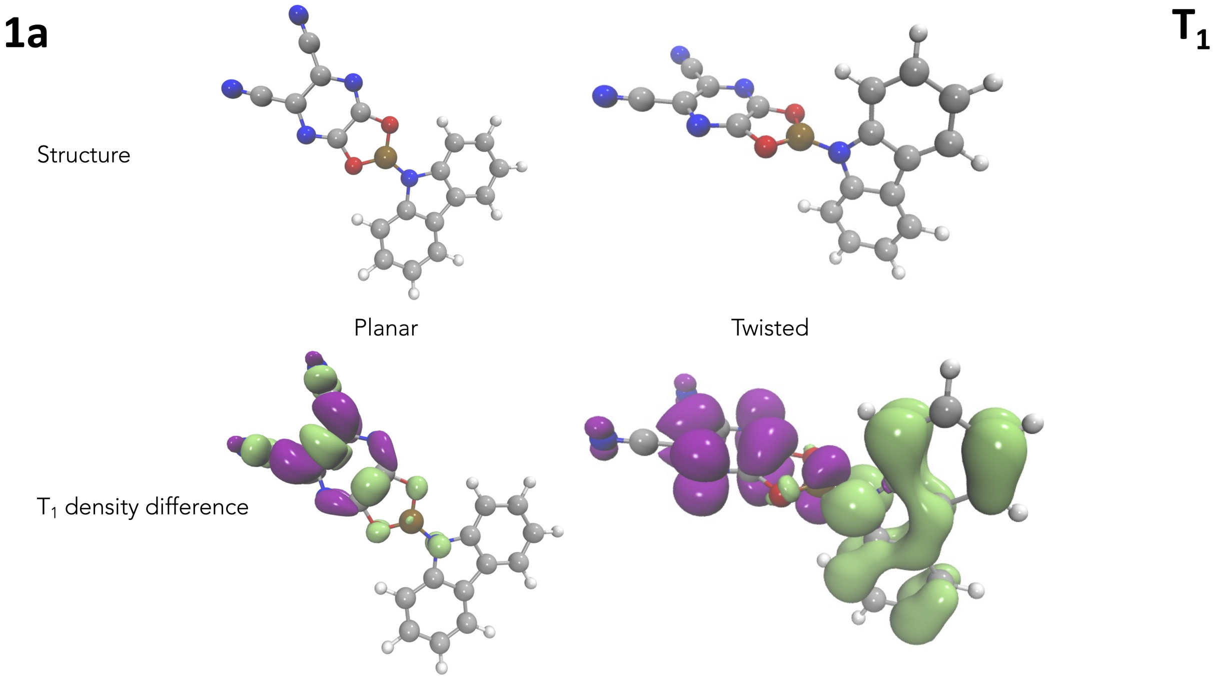

Figure S2: Structure and T_1 density difference associated with the T_1 state optimised structure of **1a**.

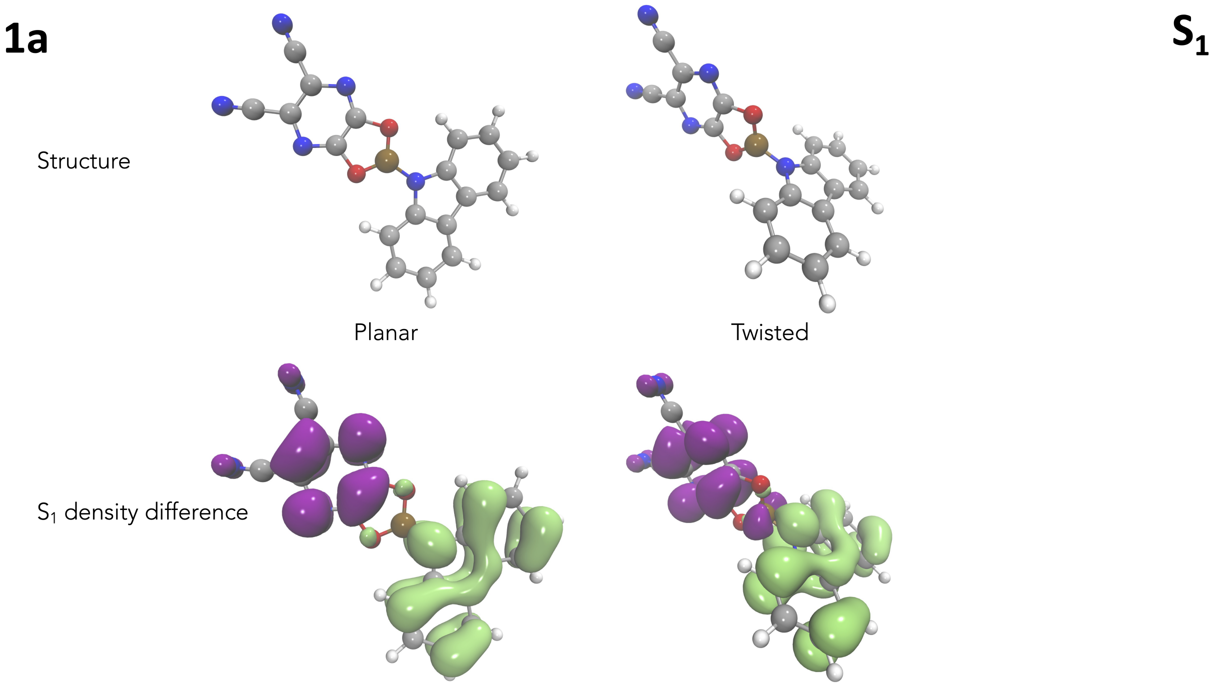

Figure S3: Structure and S_1 density difference associated with the S_1 state optimised structure of **1a**.

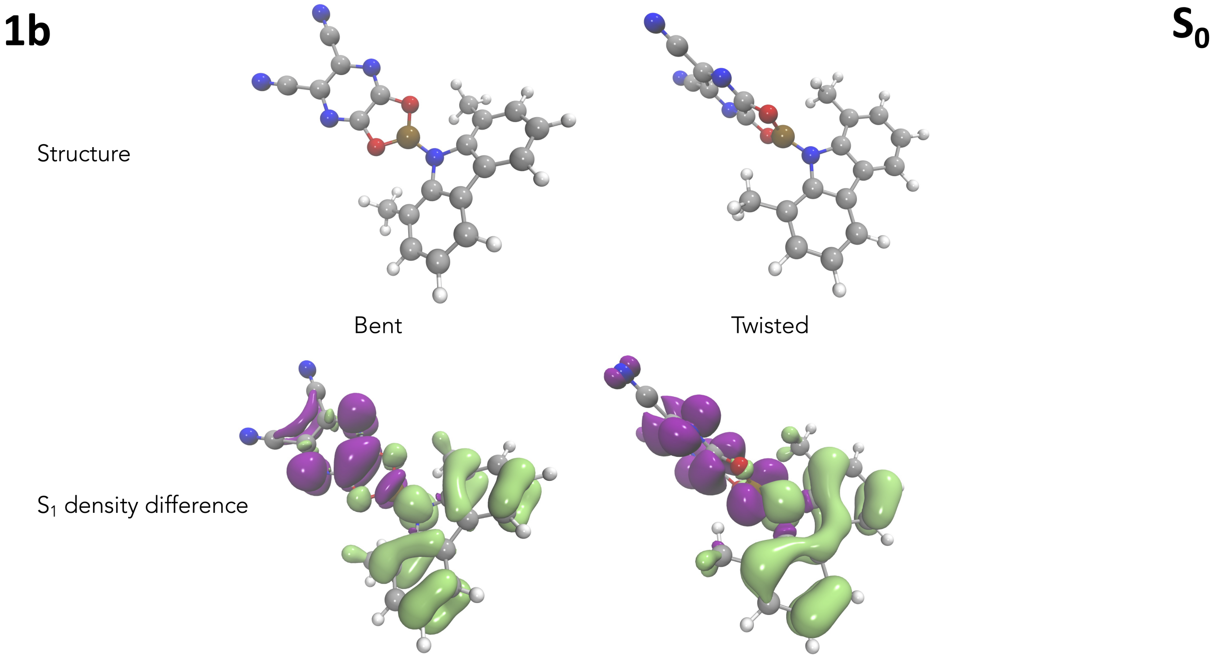

Figure S4: Structure and S_1 density difference associated with the ground state optimised structure of **1b**.

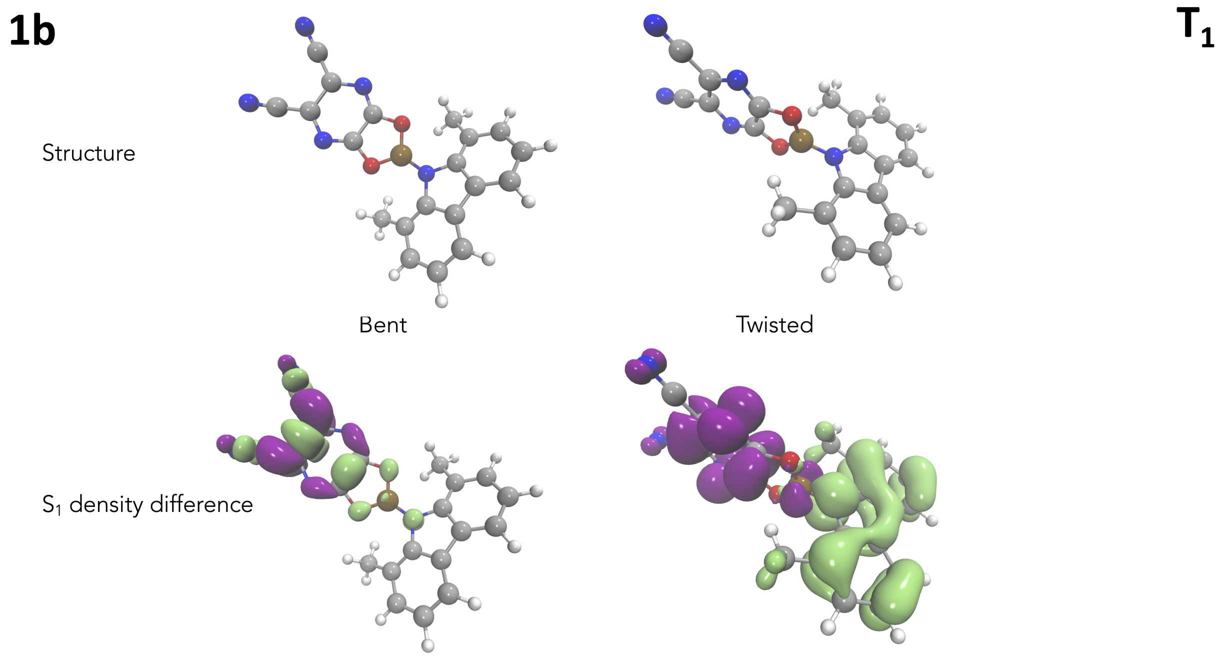

Figure S5: Structure and T_1 density difference associated with the T_1 state optimised structure of **1b**.

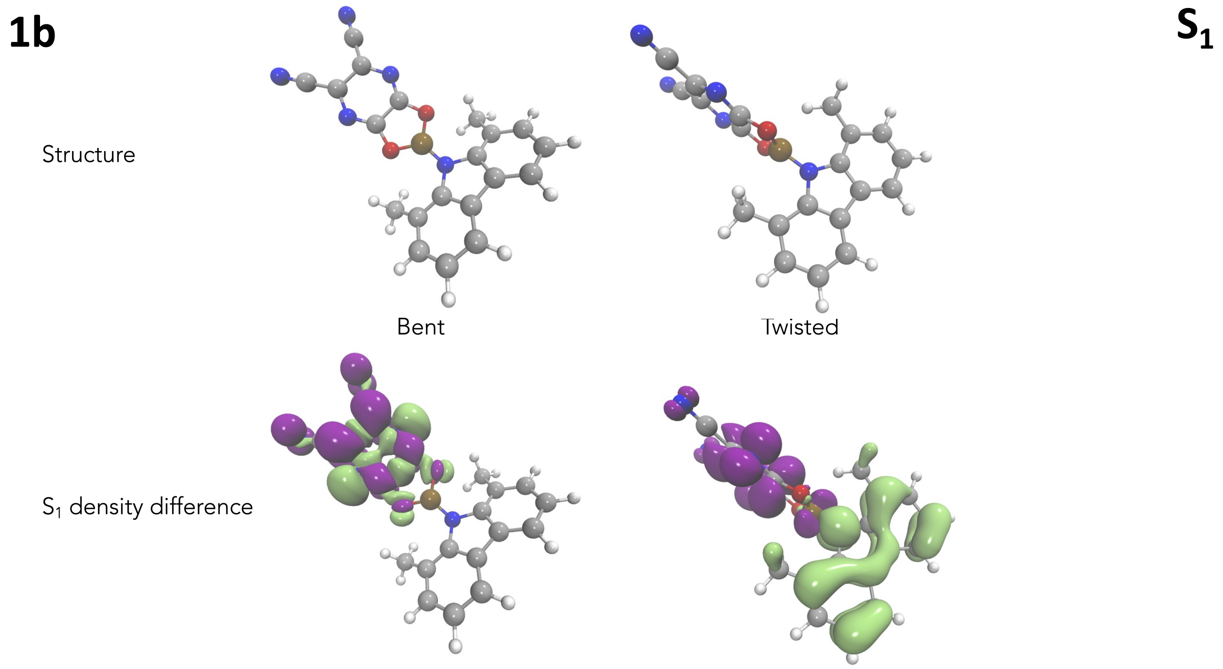

Figure S6: Structure and S_1 density difference associated with the S_1 state optimised structure of **1b**.

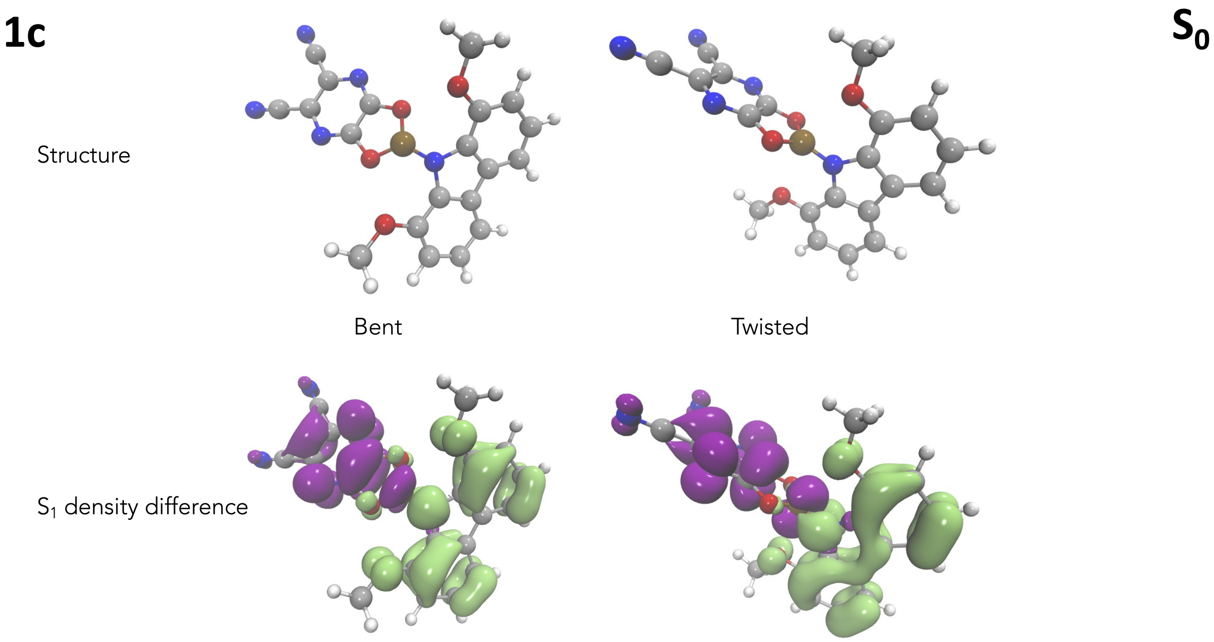

Figure S7: Structure and S_1 density difference associated with the ground state optimised structure of **1c**.

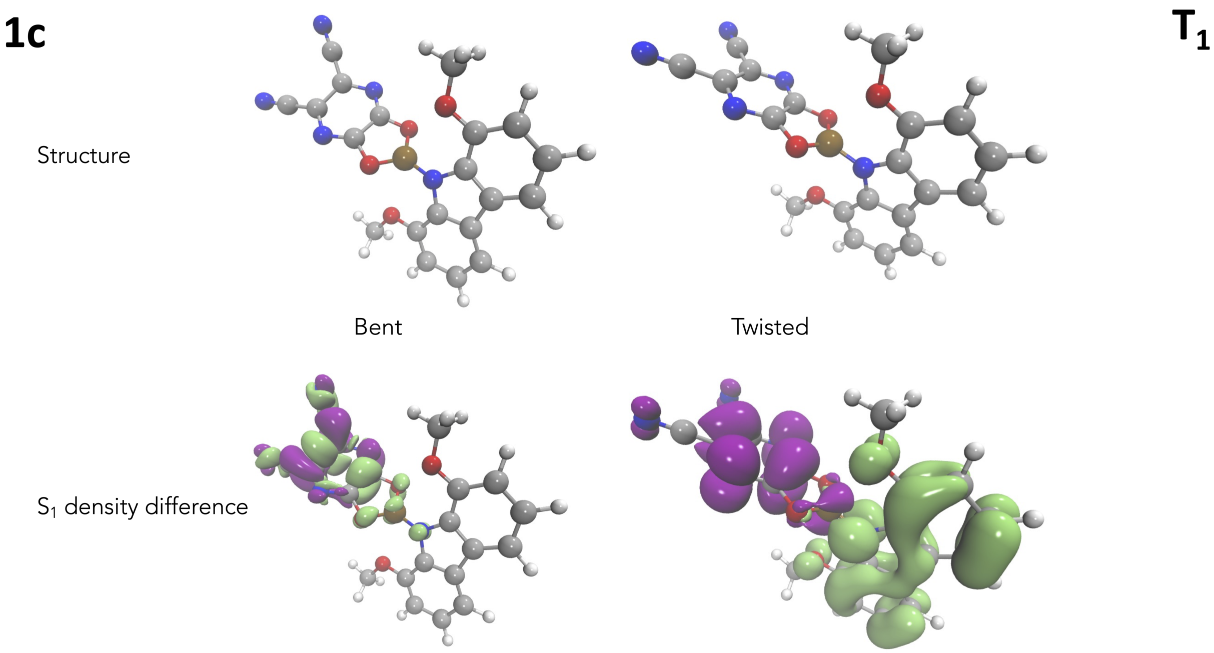

Figure S8: Structure and T_1 density difference associated with the T_1 state optimised structure of **1c**.

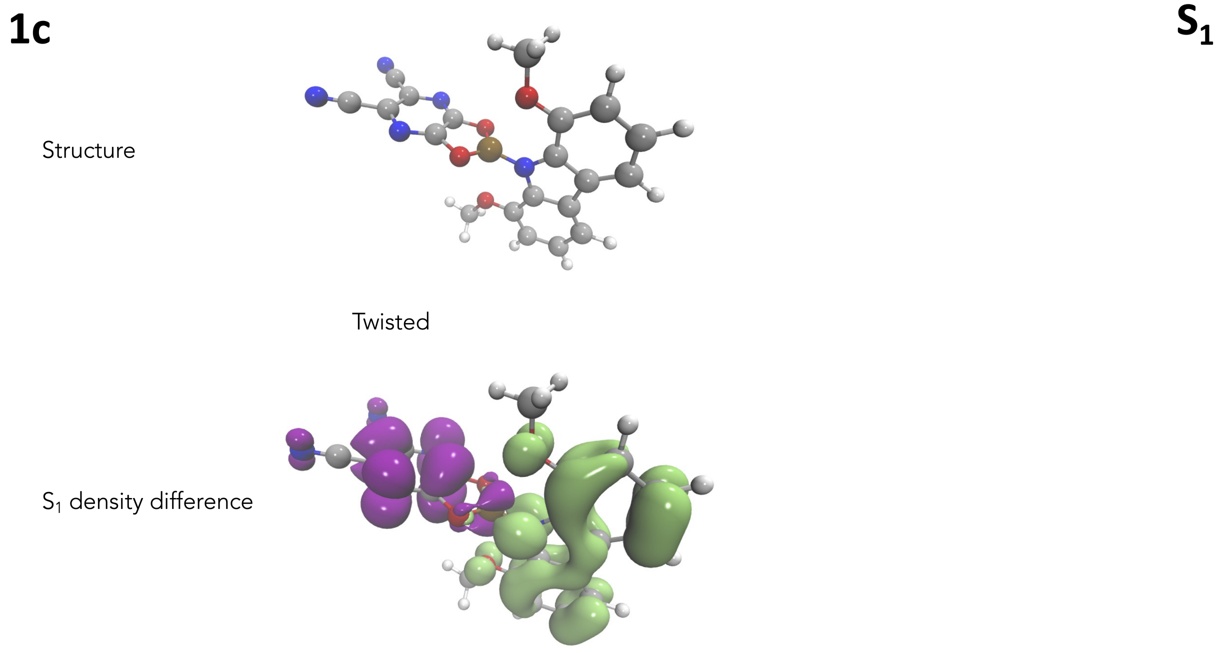

Figure S9: Structure and S_1 density difference associated with the S_1 state optimised structure of **1c**.

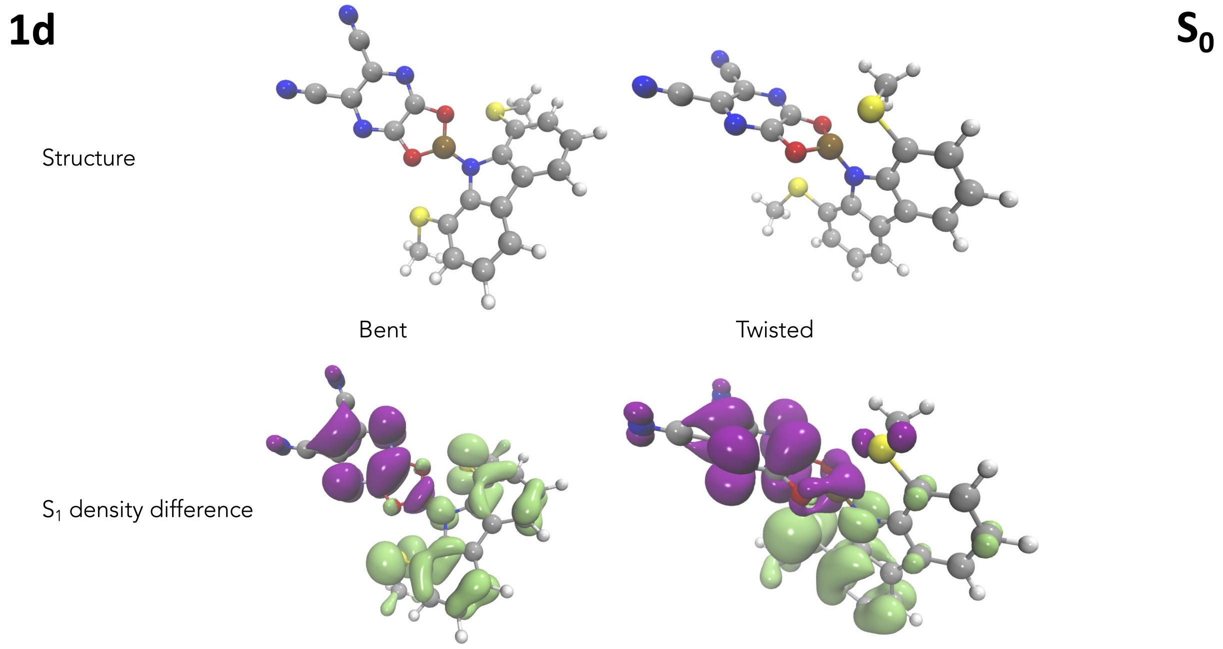

Figure S10: Structure and S_1 density difference associated with the ground state optimised structure of **1d**.

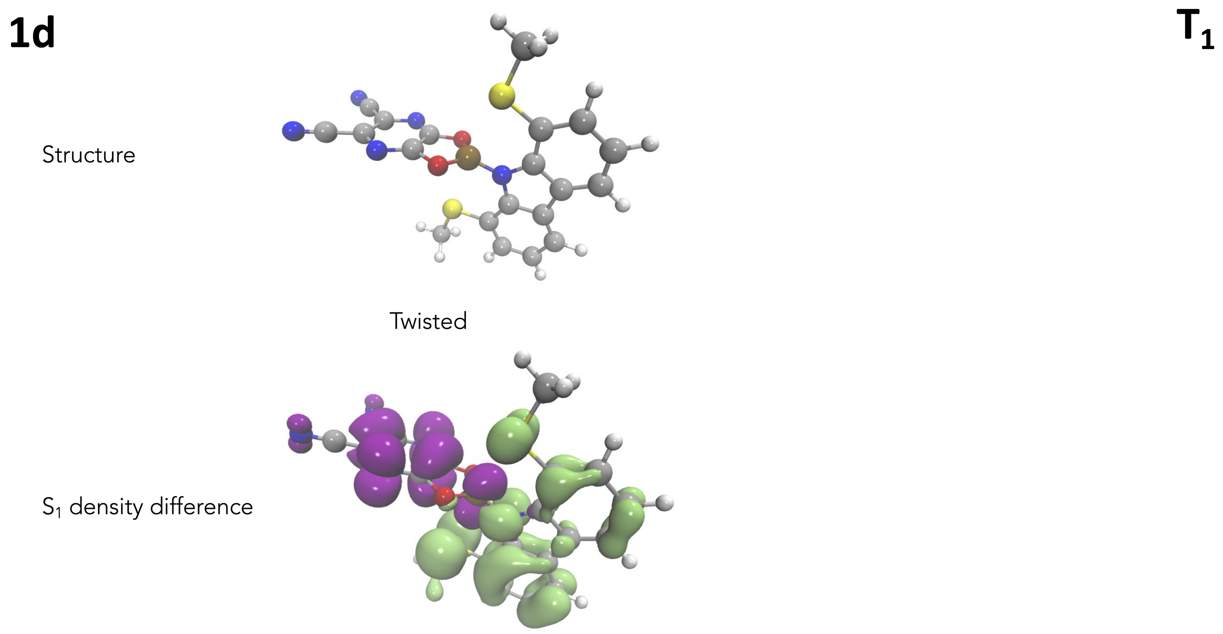

Figure S11: Structure and T_1 density difference associated with the T_1 state optimised structure of **1d**.

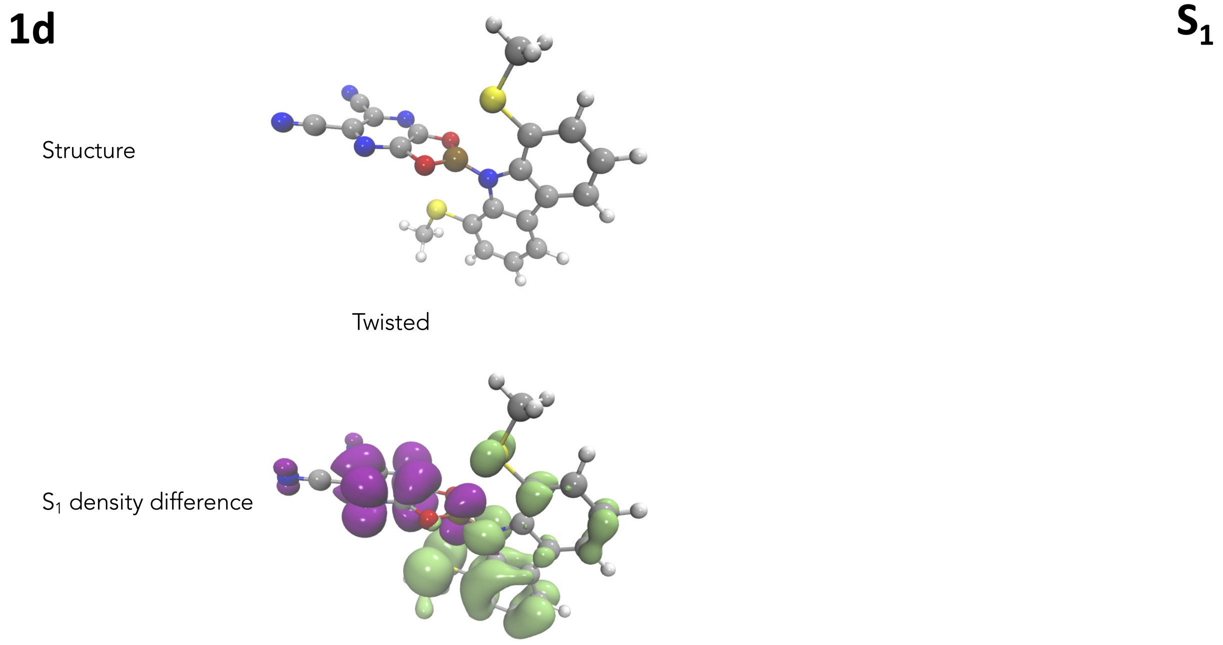

Figure S12: Structure and S_1 density difference associated with the S_1 state optimised structure of **1d**.

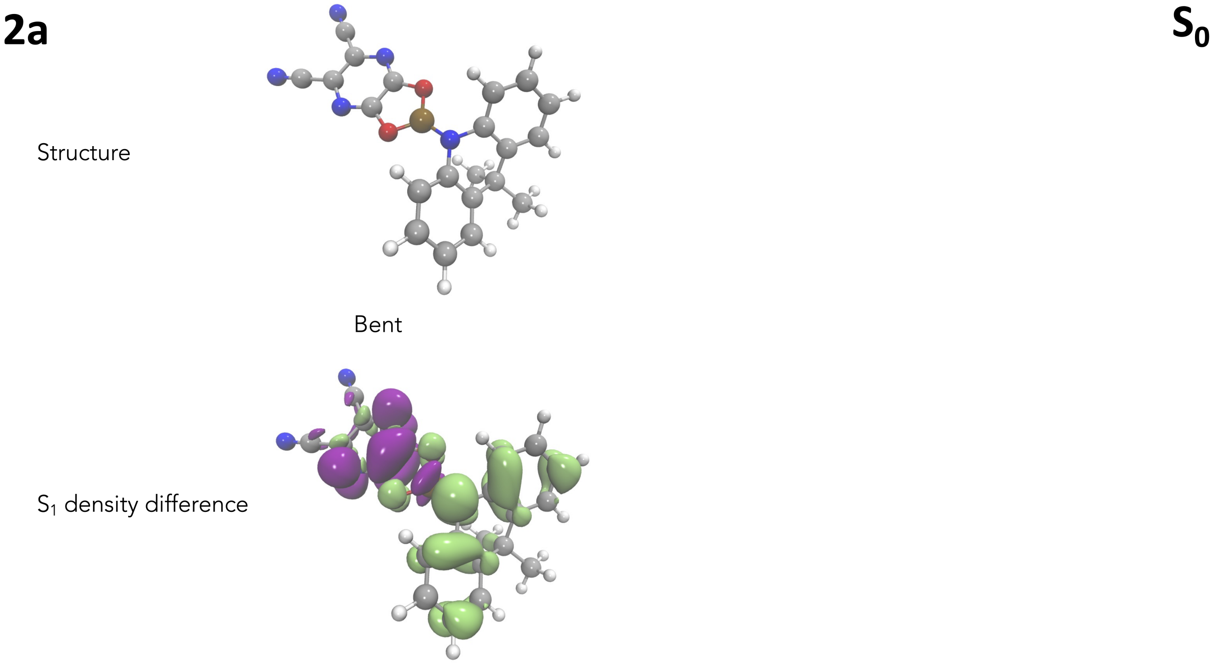

Figure S13: Structure and S_1 density difference associated with the ground state optimised structure of **2a**.

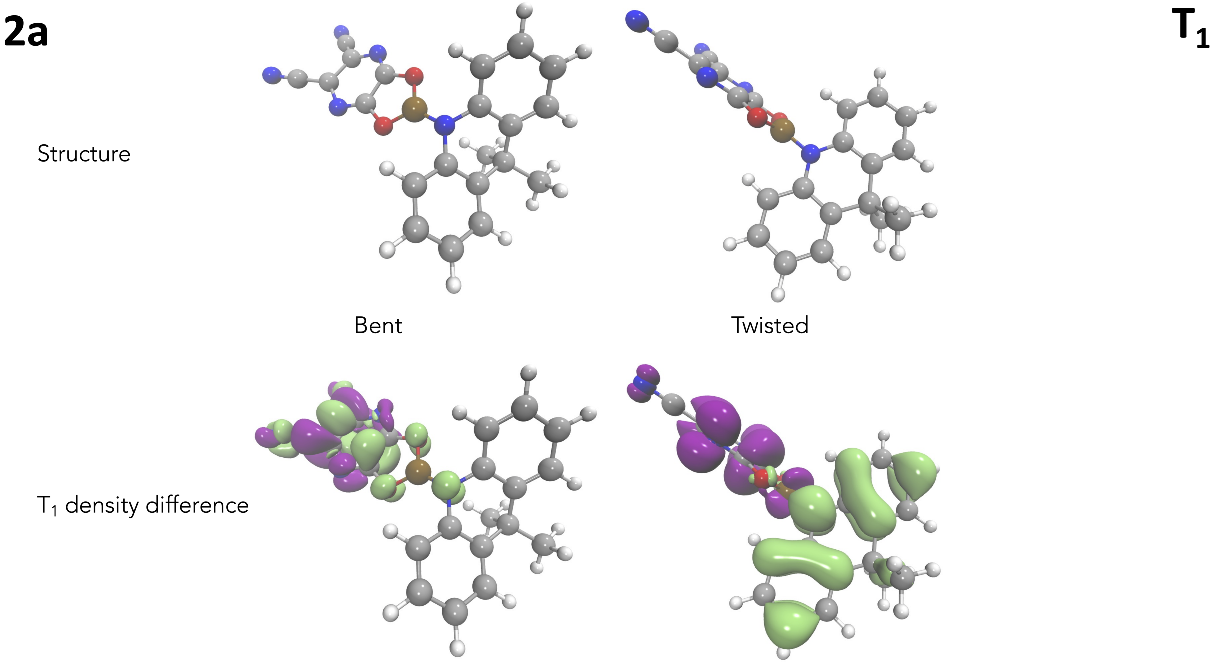

Figure S14: Structure and T_1 density difference associated with the T_1 state optimised structure of **2a**.

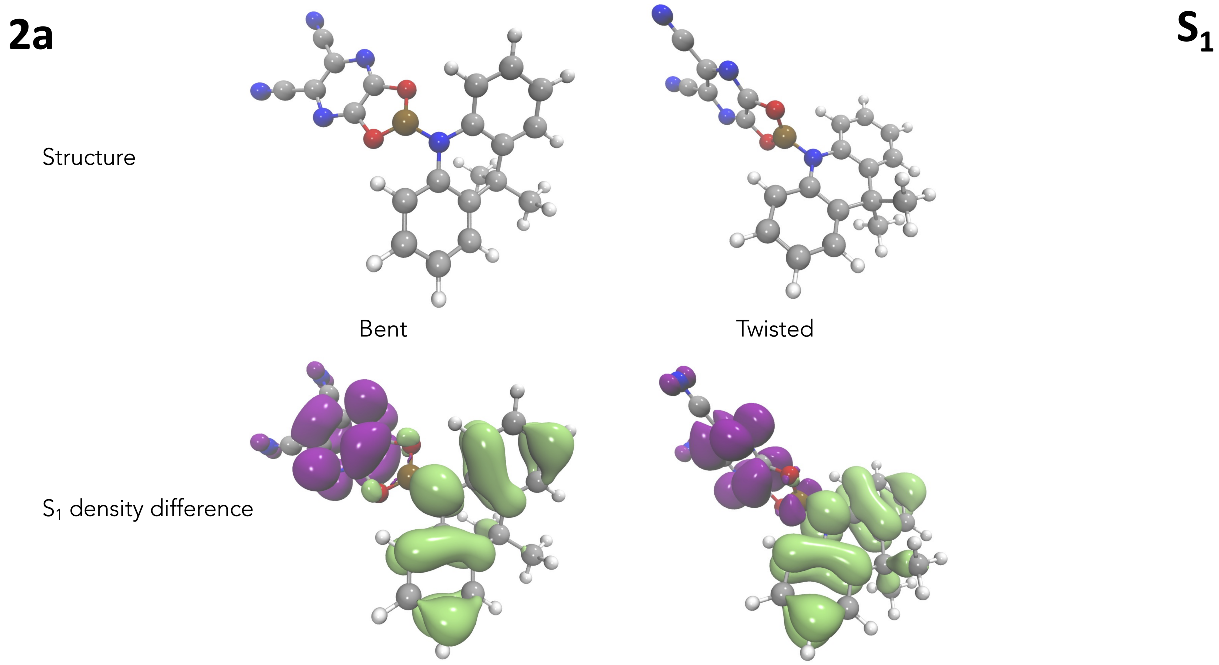

Figure S15: Structure and S_1 density difference associated with the S_1 state optimised structure of **2a**.

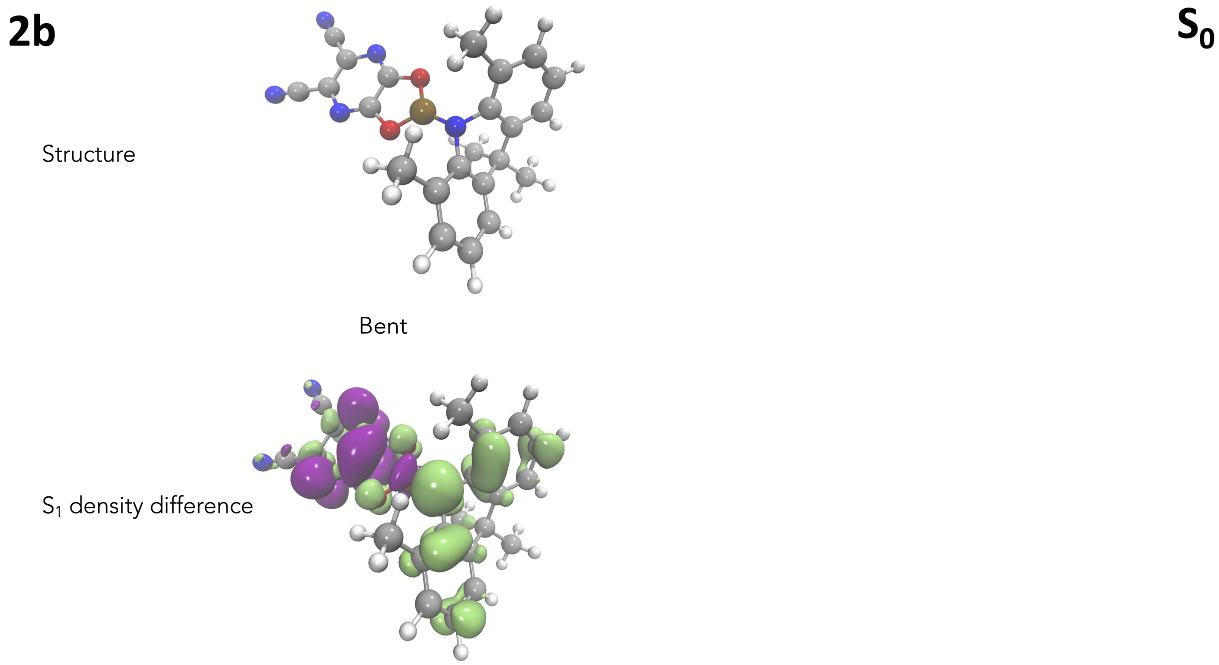

Figure S16: Structure and S_1 density difference associated with the ground state optimised structure of **2b**.

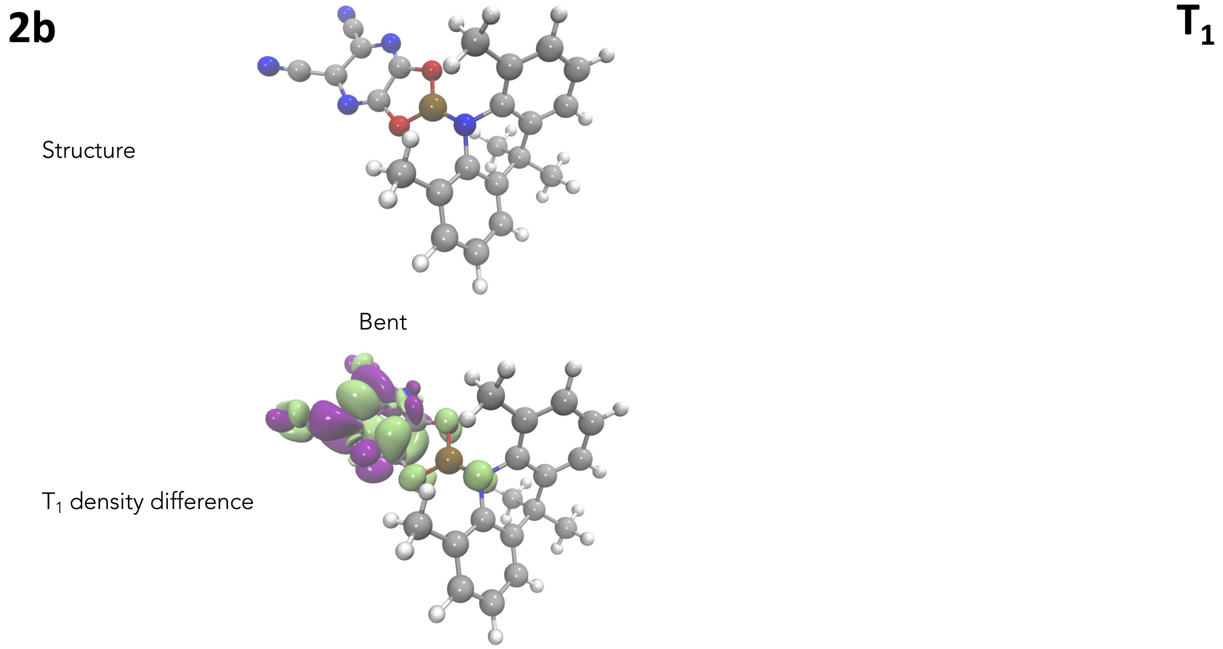

Figure S17: Structure and T_1 density difference associated with the T_1 state optimised structure of **2b**.

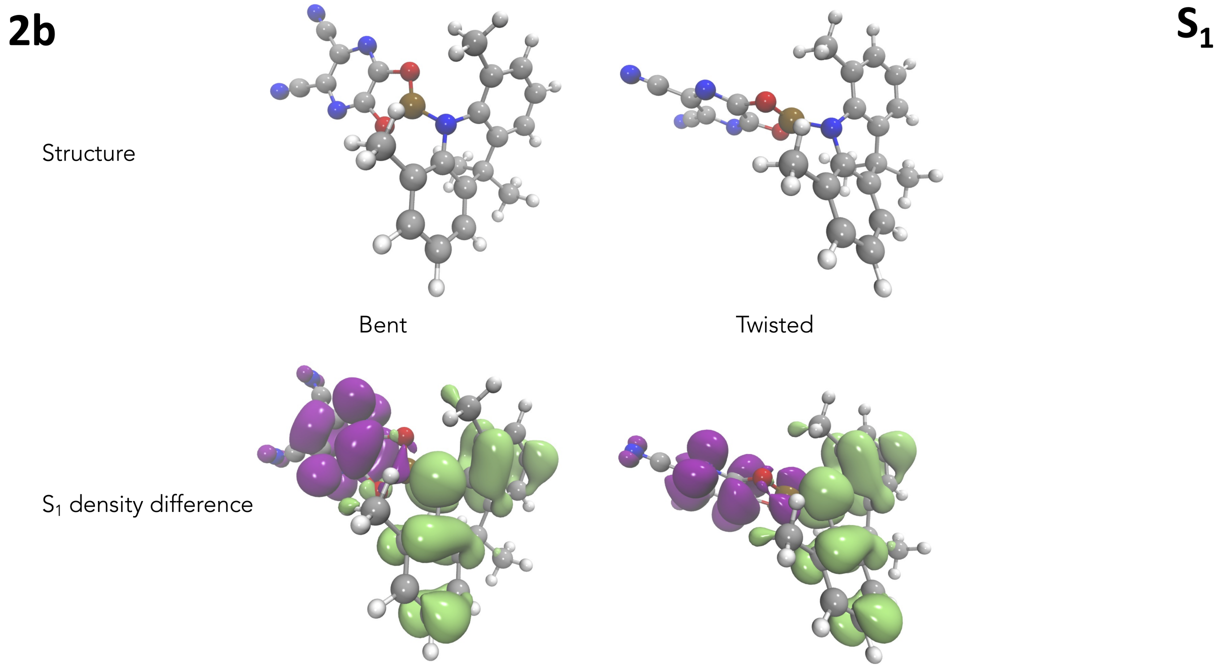

Figure S18: Structure and S_1 density difference associated with the S_1 state optimised structure of **2b**.

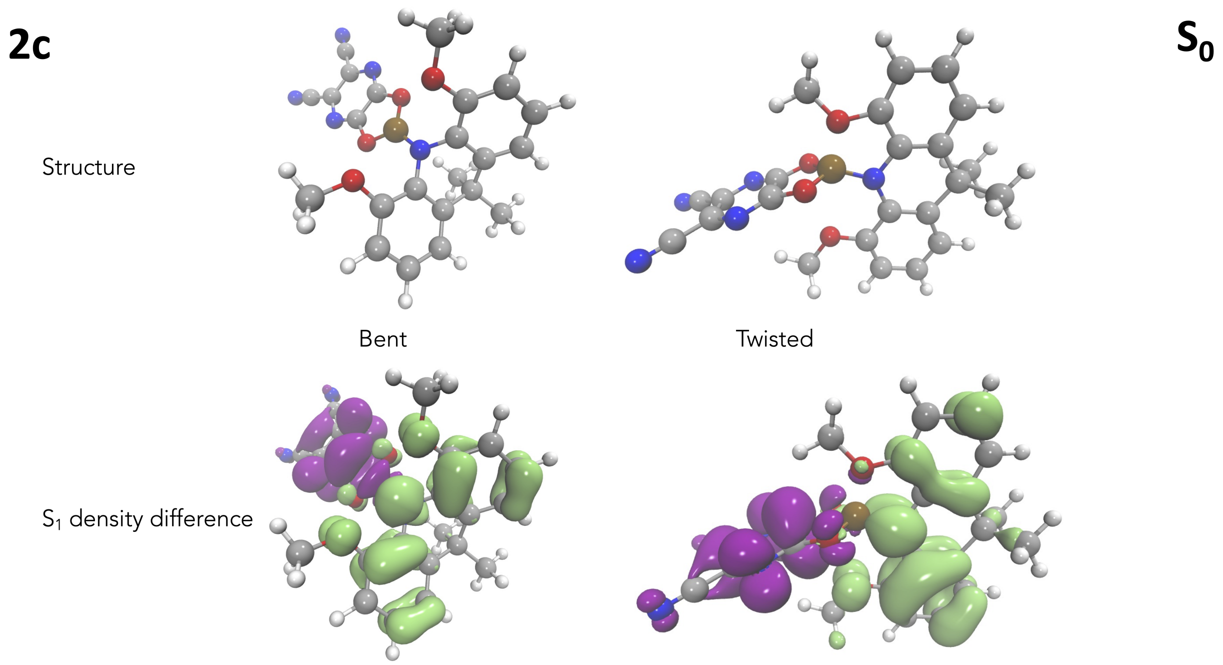

Figure S19: Structure and S_1 density difference associated with the ground state optimised structure of **2c**.

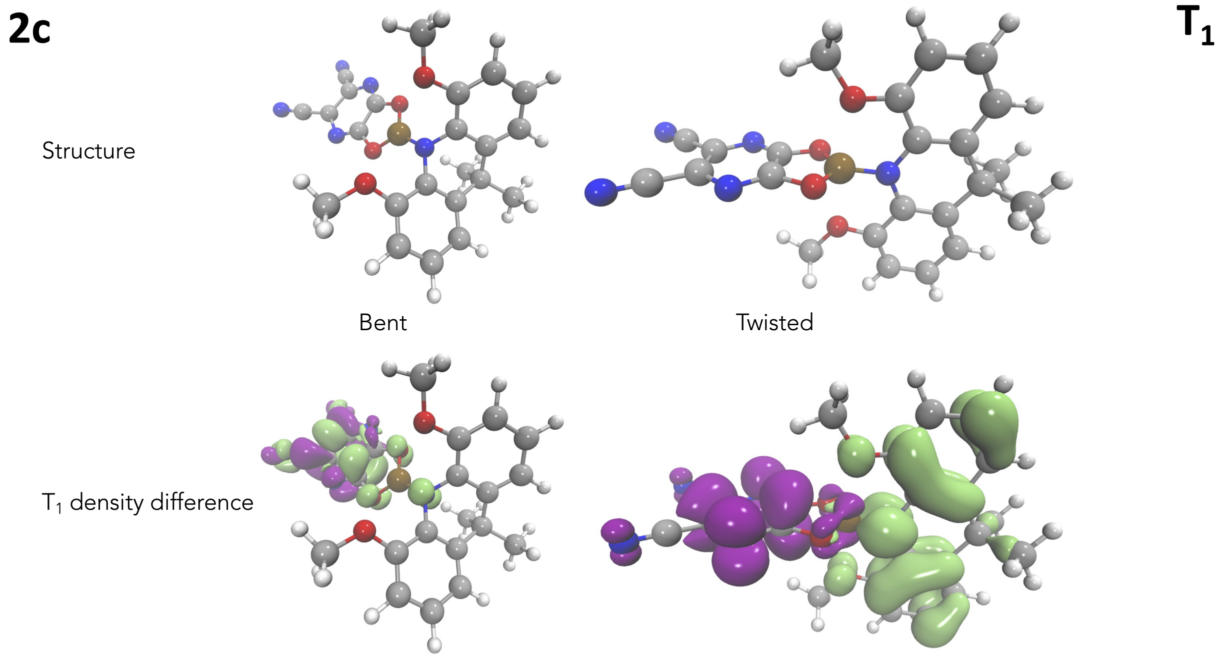

Figure S20: Structure and T_1 density difference associated with the T_1 state optimised structure of **2c**.

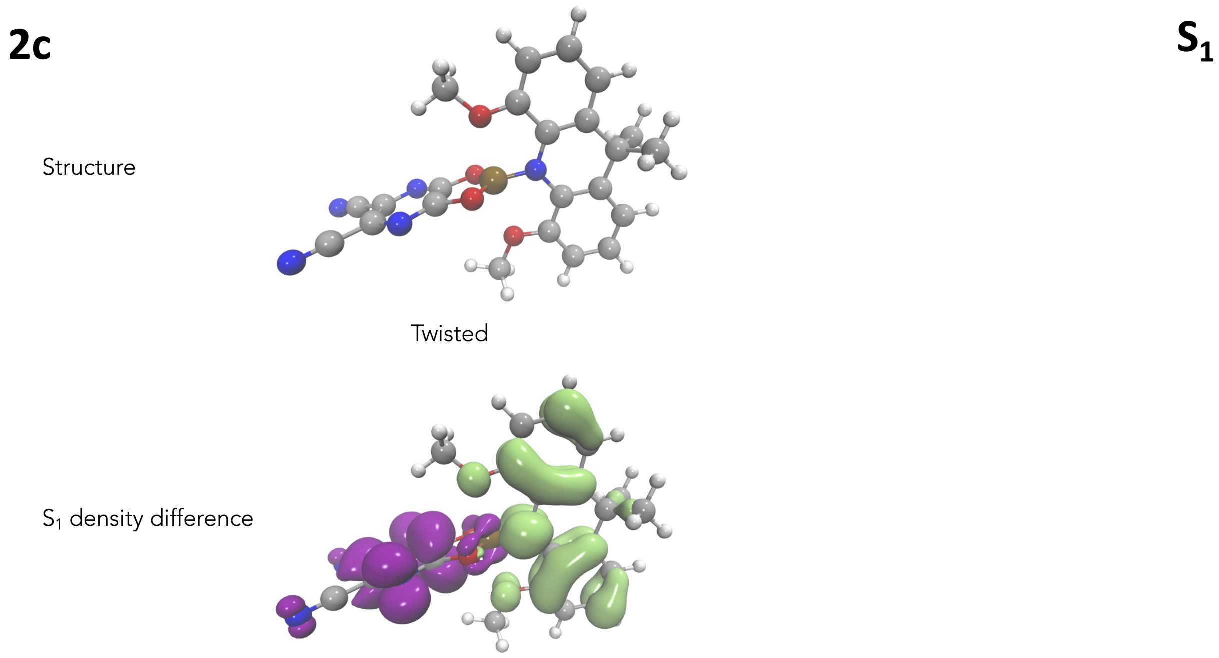

Figure S21: Structure and S_1 density difference associated with the S_1 state optimised structure of **2c**.

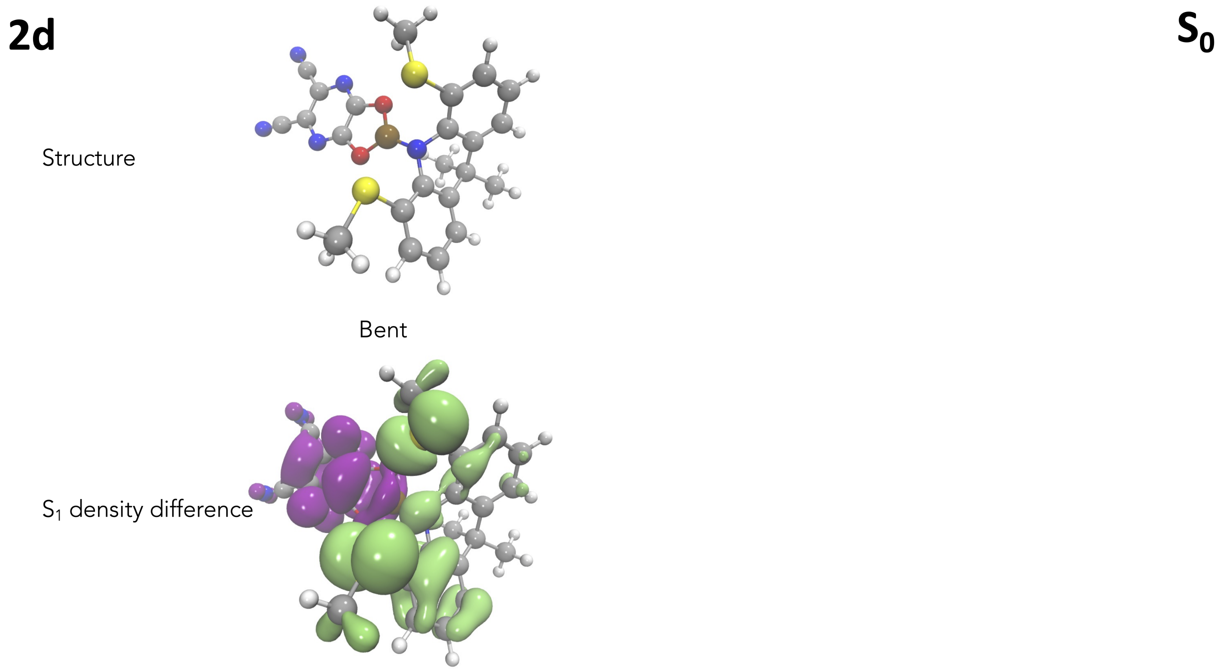

Figure S22: Structure and S_1 density difference associated with the ground state optimised structure of **2d**.

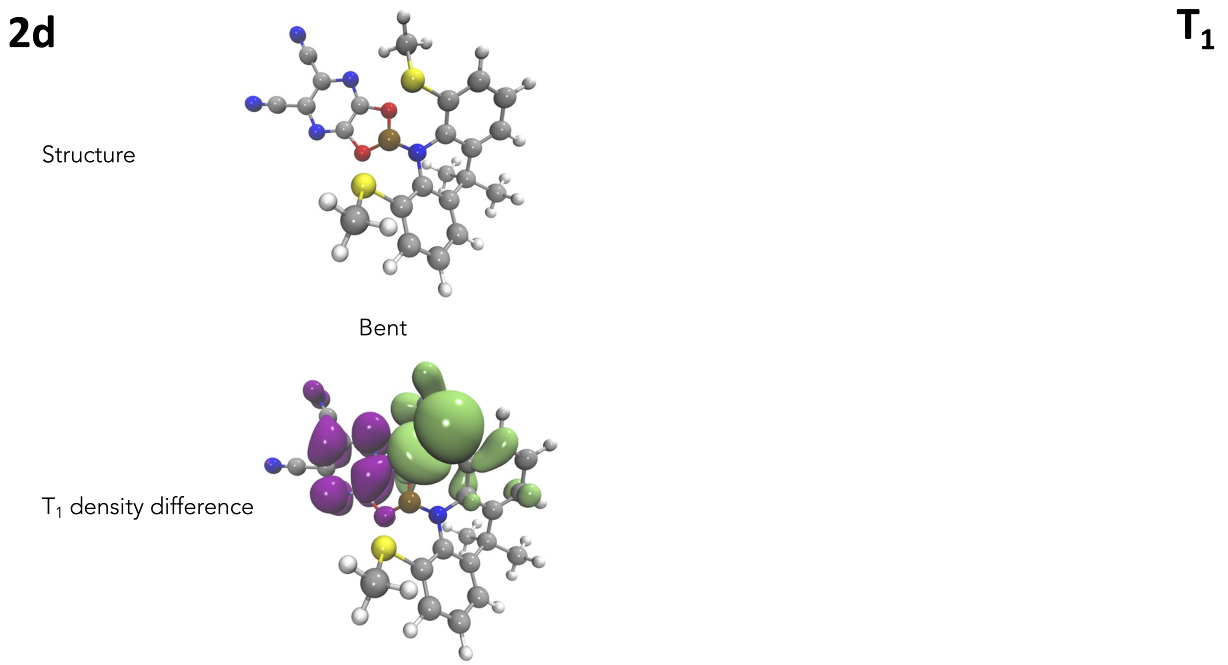

Figure S23: Structure and T_1 density difference associated with the T_1 state optimised structure of **2d**.

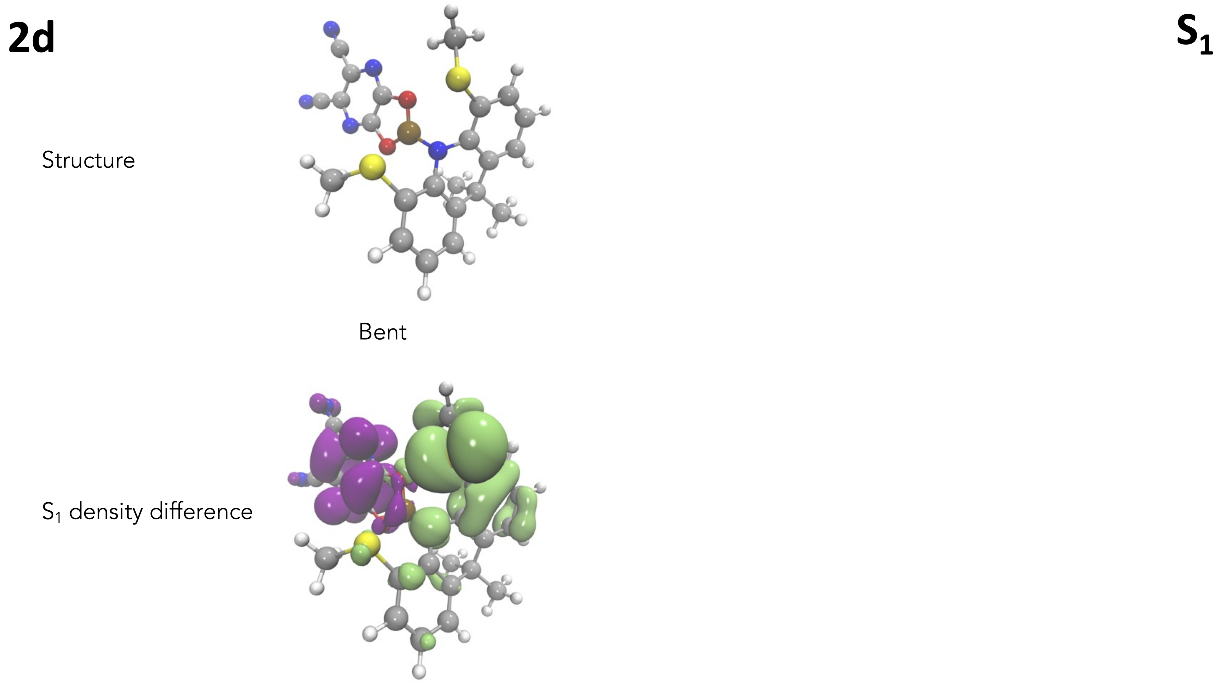

Figure S24: Structure and S_1 density difference associated with the S_1 state optimised structure of **2d**.

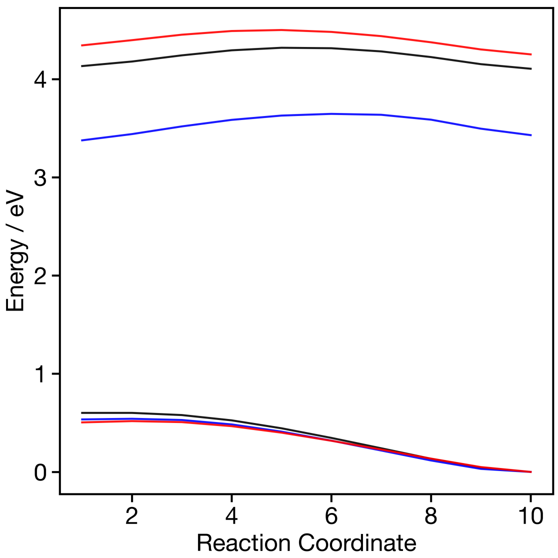

Figure S25: Potential of the ground and excited state along a linear interpolations in internal coordinates (LIICs) between the twisted and planar ground state optimised structures of **1a**. Blue is calculated using the PBE0 functional, black with the optimally tuned LC-BLYP and red with the optimally tuned ωB97X-D4 exchange and correlation functional.

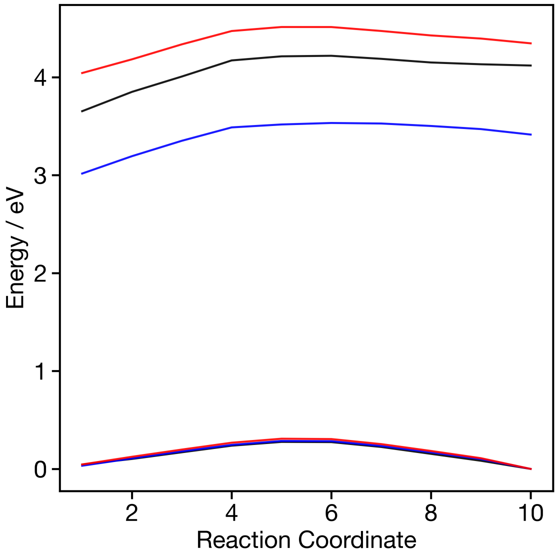

Figure S26: Potential of the ground and excited state along a linear interpolations in internal coordinates (LIICs) between the twisted and bent ground state optimised structures of **1b**. Blue is calculated using the PBE0 functional, black with the optimally tuned LC-BLYP and red with the optimally tuned ωB97X-D4 exchange and correlation functional.

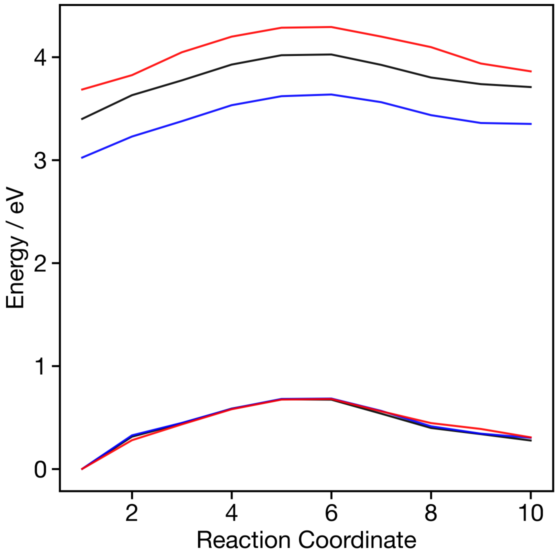

Figure S27: Potential of the ground and excited state along a linear interpolations in internal coordinates (LIICs) between the twisted and bent ground state optimised structures of **1c**. Blue is calculated using the PBE0 functional, black with the optimally tuned LC-BLYP and red with the optimally tuned ωB97X-D4 exchange and correlation functional.

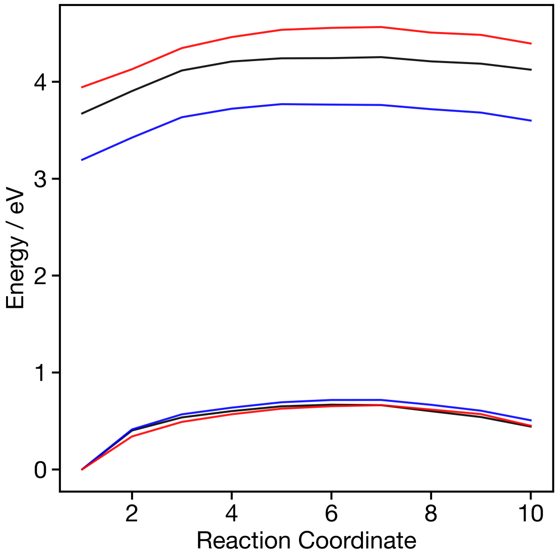

Figure S28: Potential of the ground and excited state along a linear interpolations in internal coordinates (LIICs) between the twisted and bent ground state optimised structures of **1d**. Blue is calculated using the PBE0 functional, black with the optimally tuned LC-BLYP and red with the optimally tuned ωB97X-D4 exchange and correlation functional.

	S_0 State								
	B-N / Å	B-X / Å	$\angle_\phi$ / °	$\angle_\tau$ / °	E_{S_1} / eV	E_{T_1} / eV	ΔE / eV	f_{S1}	SOC / cm^{-1}
**1a**	1.41±0.03	–	92.8±78.9	0.4±11.3	4.00±0.09	3.10±0.10	0.90±0.12	0.43±0.13	0.58±0.52
**1b**	1.42±0.03	3.19±0.17	110.7±30.1	11.7±23.1	3.70±0.20	3.11±0.13	0.59±0.25	0.23±0.13	0.56±0.39
**1c**	1.44±0.03	2.73±0.14	91.6±7.2	0.6±6.6	3.38±0.16	3.14±0.11	0.24±0.18	0.02±0.03	1.07±0.64
**1d**	1.46±0.03	2.97±0.23	90.1±14.7	14.0±10.1	3.64±0.17	3.05±0.11	0.60±0.20	0.06±0.07	2.48±1.99
**2a**	1.40±0.02	–	89.5±45.6	18.7±38.3	3.83±0.50	3.02±0.25	0.90±0.14	0.36±0.15	0.53±0.40
**2b**	1.42±0.03	2.97±0.23	99.4±20.4	42.5±25.5	3.99±0.23	3.04±0.12	0.96±0.25	0.28±0.14	0.96±0.61
**2c**	1.49±0.03	2.23±0.43	91.0±10.7	1.7±11.4	3.58±0.18	3..03±0.12	0.56±0.22	0.009±0.01	1.90±1.30
**2d**	1.50±0.03	2.67±0.40	87.9±18.6	0.1±7.0	3.56±0.15	2.94±0.12	0.61±0.19	0.035±0.038	3.06±2.37

	S_1 State								
	B-N / Å	B-X / Å	$\angle_\phi$ / °	$\angle_\tau$ / °	E_{S_1} / eV	E_{T_1} / eV	ΔE / eV	f_{S1}	SOC / cm^{-1}
**1a**	1.50±0.03	–	91.1±60.7	1.6±8.7	3.11±0.17	2.71±0.16	0.40±0.20	0.07±0.05	0.36±0.19
**1b**	1.51±0.03	3.02±0.11	85.1±6.8	0.2±4.6	2.61±0.13	2.60±0.12	0.02±0.01	0.004±0.005	0.08±0.09
**1c**	1.52±0.03	2.78±0.15	93.6±6.8	0.8±5.0	2.40±0.17	2.39±0.17	0.01±0.01	0.004±0.004	0.15±0.27
**1d**	1.52±0.04	3.02±0.28	97.0±12.3	7.8±6.6	2.44±0.18	2.41±0.17	0.03±0.06	0.003±0.03	0.75±1.07
**2a**	1.53±0.03	–	0.3±8.8	4.5±14.5	2.30±0.13	2.29±0.12	0.02±0.02	0.005±0.007	0.05±0.06
**2b**	1.53±0.03	2.97±0.23	86.1±5.6	26.9±22.4	2.46±0.19	2.40±0.15	0.06±0.06	0.015±0.015	0.21±0.18
**2c**	1.55±0.04	2.34±0.13	91.6±9.5	1.6±6.2	2.23±0.17	2.22±0.16	0.01±0.02	0.003±0.002	0.27±0.48
**2d**	1.55±0.04	2.73±0.25	92.6±23.7	0.4±8.0	2.22±0.25	2.18±0.22	0.04±0.05	0.0069±0.01	0.88±0.99

Table S16: Analysis of the geometric and electronic structure properties obtained from 15 ps of *ab initio* molecular dynamics in the electronic ground (S_0) and first singlet excited state (S_1).
